# PIEZO1-mediated calcium signaling reinforces mechanical properties of hair follicle stem cells to promote quiescence

**DOI:** 10.1126/sciadv.adt2771

**Published:** 2025-05-28

**Authors:** Jingjing Wang, Chaoyu Fu, Sophie Chang, Christopher Stephens, Haimin Li, Dongmei Wang, Yuheng C. Fu, Kathleen J. Green, Jie Yan, Rui Yi

**Affiliations:** ^1^ Department of Pathology, Northwestern University Feinberg School of Medicine, Chicago, IL 60611 USA.; ^2^ Department of Physics, National University of Singapore, Singapore 117551, Singapore.; ^3^ Mechanobiology Institute, National University of Singapore, Singapore 117411, Singapore.; ^4^ Driskill Graduate Program in Life Sciences, Northwestern University Feinberg School of Medicine, Chicago, IL 60611 USA.; ^5^ Department of Dermatology, Northwestern University Feinberg School of Medicine, Chicago, IL 60611 USA.; ^6^ Robert H. Lurie Comprehensive Cancer Center, Northwestern University Feinberg School of Medicine, Chicago, IL 60611 USA.

## Abstract

The mechanisms by which epithelial stem cells (SCs) sense mechanical cues within their niche and convert the information into biochemical signals to govern their function are not well understood. Here, we show that hair follicle SCs (HF-SCs) sense mechanical forces through cell adhesion and maintain quiescence in a PIEZO1-dependent mechanism. PIEZO1 interacts with E-cadherin in HF-SCs, and mechanical pulling of E-cadherin with a force of ~20 pN triggers PIEZO1-dependent, localized calcium flickers. Deletion of *Piezo1* leads to reduced cumulative calcium influx and compromises quiescence. Single-cell genomic analyses identify a transcriptional network involving AP1 and NFATC1, which functions downstream of PIEZO1 and regulates the expression of extracellular matrix, cell adhesion, and actin cytoskeleton genes to reinforce the unique mechanical property of HF-SCs. These findings establish the force threshold necessary for PIEZO1 activation and reveal PIEZO1-dependent calcium influx as a key mechanism for sensing mechanical cues in the niche and regulating HF-SC activity.

## Teaser

PIEZO1 senses mechanical forces between epithelial cells and controls stem cell behavior in hair follicles.

## INTRODUCTION

Tissue stem cells (SCs) sense and respond to external cues from their microenvironment, which is critical for maintaining their identity and modulating their activities necessary for homeostasis, tissue regeneration, and wound repair. Classic signal transduction pathways, such as WNT, BMP and SHH pathways, translate discrete biochemical signals into internal cellular responses, orchestrating SC functions through complex molecular cascades. These cascades typically involve distinct molecular ligands, specific receptors, and a series of intracellular effectors, including protein modification enzymes and transcription factors (TFs). Recent studies have also revealed how physical properties of SC microenvironment, such as mechanical forces and the stiffness of extracellular matrix (ECM), exert a continuous and potent influence on SC fate and behavior (*1*–*3*). These biophysical cues, unlike their molecular counterparts, regulate SC activities through more sustained and pervasive mechanisms, affecting aspects such as cell size, shape, cytoskeletal contractility, and gene expression (*4*, *5*). In turn, tissue SCs continuously adapt to these mechanical stimuli, critical for biological processes ranging from tissue regeneration (*2*) to tumor progression (*6*, *7*). Despite growing knowledge, the mechanisms through which tissue SCs integrate and translate these persistent mechanical cues into coordinated cellular responses remain incompletely understood. Among mechanotransduction pathways, calcium (Ca^2+^) signaling mediated by mechanosensitive (MS) channels has long been implicated for its role in dynamically regulating cellular responses to mechanical stimuli (*4*, *8*, *9*). However, multiple mechanisms, including Ca^2+^ release and uptake by the endoplasmic reticulum and mitochondria, could regulate cytosolic Ca^2+^ levels in response to stress, making it challenging to study Ca^2+^ signaling in mechanosensing. Furthermore, the specific mechanisms through which SCs translate transient Ca^2+^ influx signals to continuously control cellular states within their niche remain unclear (*8*).

Hair follicle SCs (HF-SCs) and their closely related hair germ (HG) progenitors provide an experimental system to examine molecular mechanisms underpinning tissue SC maintenance and activation in a spatiotemporally specific manner (*10*–*14*). Recent application of two-photon live imaging to HF-SCs has opened a frontier in tissue SC biology to monitor cell signaling events, including Ca^2+^ signaling (*15*), and cellular activities in their intact microenvironment (*12*). We and others applied atomic force microscopy (AFM) to measure the stiffness of HF-SC and HG compartments and revealed a spatiotemporally dynamic pattern of tissue rigidity of the HF-SC compartment during the quiescent telogen and the telogen-to-anagen transition and aging (*14*, *16*). Unlike the epidermis and the crypts in the intestine, where proliferative SCs reside in a relatively soft microenvironment and differentiated daughter cells are located in a stiff microenvironment (*6*, *17*, *18*), bulge HF-SC compartment is stiff, whereas the HG is relatively soft. We have further demonstrated that manipulation of these mechanical properties of HF-SCs by reducing the stiffness and actomyosin contractility promotes hair regeneration (*14*). However, how HF-SCs sense the mechanical properties of their microenvironment and maintain their stiffness remains unclear.

In this study, we perform intravital Ca^2+^ imaging and identify two different types of Ca^2+^ spikes based on the amplitude quantified by a ratiometric Ca^2+^ reporter (*19*) in vivo. Notably, the relatively weak but pervasive Ca^2+^ influx in bulge HF-SCs is controlled by the MS PIEZO1 channel (*20*). We further demonstrate that E-cadherin interacts with PIEZO1 in bulge HF-SCs. Notably, mechanical pulling E-cadherin–coated microbeads with ~20-pN force is sufficient to activate PIEZO1 and triggers localized Ca^2+^ flickers in primary keratinocytes, recapitulating PIEZO1-depedent Ca^2+^ influx observed in bulge HF-SCs. Genetic and genomic studies have revealed the mechanism of mechanical sensing in bulge HF-SCs through a PIEZO1-Ca^2+^ influx–transcription factor (TF) cascade and provided a mechanistic insight into how HF-SCs leverage PIEZO1-mediated Ca^2+^ influx to reinforce their unique mechanical microenvironment and maintain SC cell states.

## RESULTS

### Spatiotemporal dynamics of calcium influx are detected in HF-SC compartment

**Fig. 1.**
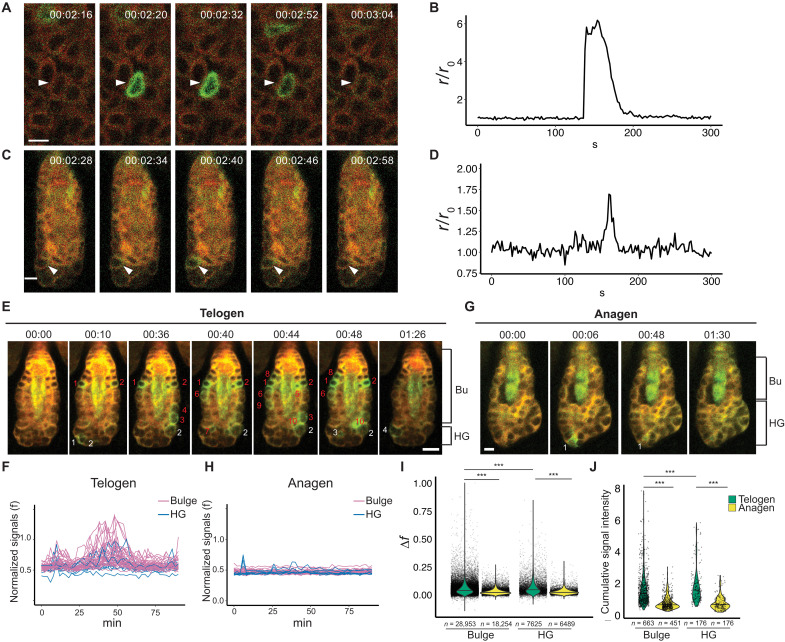
HF-SC compartments display differential calcium dynamics during quiescence and activation. (**A**) High-intensity calcium spike (arrowhead) recorded in the epidermis. Each image is annotated with the elapsed time from the start of the experiment. Scale bar, 20 μm. (**B**) Quantification of the calcium spike recorded in (A). The graph shows the fold change in the ratio of GCaMP6f to tdTomato fluorescence $\left(\frac{r}{r_{0}}\right)$ as a function of time. (**C**) Calcium spike (arrowhead) recorded in bulge HF-SCs. Each image is annotated as in (A). Scale bar, 20 μm. (**D**) Quantification of the calcium spike observed in (C) in the same manner as in (B). (**E** and **G**) Calcium spikes (numbered) observed in the bulge (Bu) and HG compartments in telogen (E) and anagen (G). Red numbers indicate bulge cells with calcium spike events, and white numbers indicate HG cells with calcium spike events. Identical HFs with more time points are shown in fig. S4. Scale bar, 10 μm. (**F** and **H**) Normalized calcium signals (f) of all HF-SCs and HG cells in telogen (F) over 90 min and in anagen (H) over 90 min. Each purple line represents calcium signals for an HF-SC cell. Blue lines show calcium signals for HG cells. (**I**) Calcium signal change (Δ*f*) of bulge HF-SC and HG cells in telogen and anagen. A total of 663 bulge cells and 176 HG cells of 17 telogen HFs, 451 bulge cells of 14 anagen HFs, and 176 HG cells of 12 anagen HFs, from four animals, are quantified. Total signal events are indicated by *n* numbers in the panel. Statistical significance was determined by Mann-Whitney *U* test (***P < 0.001). (**J**) Cumulative calcium signals of bulge HF-SC and HG cells are reduced in anagen compared to telogen. Statistical significance was determined by Mann-Whitney *U* test (***P < 0.001).

To determine whether Ca^2+^ influx plays a role in mechanical sensing of HF-SCs, we first applied a resonant scanner with a frame rate of 2 s per frame to detect individual Ca^2+^ spikes in bulge HF-SCs and the epidermis as control during the second telogen between postnatal day 42 (P42) and P70. The Ca^2+^ imaging (fig. S1) was achieved by using a *Krt14-Cre/LSL-Salsa6f* (Jax #031968) mouse model, in which tdTomato (tdT) is fused to GCaMP6f (*19*). For ratiometric Ca^2+^ analysis, the fused tdT was used as internal control for GCaMP6f signals that are dependent on Ca^2+^ binding (fig. S1, A to E) (*19*). We readily detected both individual (Fig. 1, A and B) and coordinated Ca^2+^ spikes in the epidermis (Fig. 1F), as described recently (*15*). In contrast, Ca^2+^ spikes in bulge HF-SCs were weaker (Fig. 1, C and D). Notably, Ca^2+^ spikes in the epidermis ramped up rapidly and gradually waned in the next 60 to 80 s (Fig. 1B and fig. S1, D and E), whereas Ca^2+^ spikes in HFs generally showed a lower amplitude and shorter duration (Fig. 1D).

To enhance the quality for deep tissue imaging of the HFs, we next used nondescanned detectors to perform ratiometric Ca^2+^ imaging. Because the quiescent telogen phase typically ranges from days to weeks (*21*) and epithelial cells of the skin are not excitable, we reasoned if Ca^2+^ signaling plays a role in regulating HF-SC activities, then cumulative Ca^2+^ dynamics over time, rather than individual Ca^2+^ spike events, are more likely to have a measurable impact on HF-SC states. Furthermore, because photodamage triggers Ca^2+^ waves through adenosine 5′-triphosphate (ATP) release and purinergic G protein-coupled (P2Y) receptors in the epidermis (*22*, *23*), we aimed to minimize stress and reduce the frequency of imaging while extending the length of imaging sessions. Because a single Ca^2+^ spike lasts ~50 to 100 s (Fig. 1, B and D), we recorded ratiometric Ca^2+^ signals every 2 min for a duration of 90 min per imaging session for quantifying cumulative Ca^2+^ dynamics. During these extended imaging sessions, we also used the tdT signals to control for systemic signal variations, caused by sample drift and photobleaching (fig. S2, A to E). We calculated the corrected Ca^2+^ signals by normalizing GCaMP6f signals over tdT, denoted as f (fig. S2E and see Materials and Methods). Because Ca^2+^ signals in HFs are relatively weak, we further calculated the change in Ca^2+^ signals over the background, denoted as Δf, which allows us to better detect *bona fide* Ca^2+^ spikes from the background (fig. S2F and see Materials and Methods). Consistent with the results obtained from resonant scanner, we also observed strong but transient Ca^2+^ spiking events in both individual cells and a patch of cells in the epidermis (fig. S3A and movie S1). We also confirmed that Ca^2+^ spiking events were much weaker in the HFs than their epidermal counterparts (movie S1). Within the HFs, we occasionally observed strong Ca^2+^ events in individual cells, reminiscent of the patterns observed in the interfollicular epidermis (fig. S3, A and B). However, most Ca^2+^ spikes in HF-SCs were relatively weak but recurrent (fig. S3C). Quantification of the Ca^2+^ signals (f) suggested two different types of Ca^2+^ spikes, judging by the maximum fold change over the background (f/f0) (fig. S3D). Because the high-intensity events were rare in HFs, generally less than 1 event in each 90-min imaging session, we focused our study to the low amplitude but recurrent events. We observed more Ca^2+^ spikes in bulge HF-SCs compared to HG progenitors during the telogen phase (Fig. 1E, fig. S4, A and B, and movie S2). Closer inspection revealed that both the number of Ca^2+^ spikes and the number of cells exhibiting these events were greater in HF-SCs than in HG progenitors (fig. S4, B and C). Quantification of normalized Ca^2+^ signals (f) indicated stronger signals in the bulge than HGs (Fig. 1F and fig. S4D). Transitioning to early anagen, we still observed Ca^2+^ spikes in both compartments, although the frequency, signal amplitude, and the number of cells showing Ca^2+^ spikes were all significantly reduced compared to telogen (Fig. 1, G and H; fig. S4, E and F; and movie S3).

To further characterize the dynamics in telogen and early anagen, we quantified both normalized Ca^2+^ signals (f) (fig. S4G) and Ca^2+^ signal change over the background (Δf) (Fig. 1I). For the bulge, more than 28,000 time points for hundreds of HF-SCs (663 cells) in telogen and more than 18,000 time points in early anagen (451 cells) were analyzed; for HGs, more than 7600 time points in telogen (176 cells) and more than 6400 time points in early anagen (176 cells) were analyzed. These results quantified Ca^2+^ spiking events across a large number of cells and time points and revealed stronger Ca^2+^ spikes in bulge HF-SCs versus HGs and in telogen versus early anagen. This extensive collection of time-lapse datasets also enabled us to quantify the cumulative Ca^2+^ spikes (during the course of 90 min) for individual bulge HF-SCs and HG progenitors in refractory telogen (generally synchronized ~P49) and early anagen (generally synchronized ~P70), respectively. In line with the results obtained for individual Ca^2+^ spikes, these data revealed that both cell types exhibit a greater amount of cumulative Ca^2+^ spikes in telogen than early anagen (Fig. 1J). The pattern of cumulative Ca^2+^ dynamics in telogen and early anagen suggests a correlation between Ca^2+^ and the quiescence and activation of HF-SCs. Furthermore, these variations in Ca^2+^ spikes are also correlated with the changes in mechanical properties, such as stiffness and actomyosin contractility, which we have measured recently (*14*). These observations prompted us to test the hypothesis that Ca^2+^ dynamics links mechanical properties and the quiescence of HF-SCs.

### PIEZO1 mediates dynamic calcium influx in HF-SC compartment

**Fig. 2.**
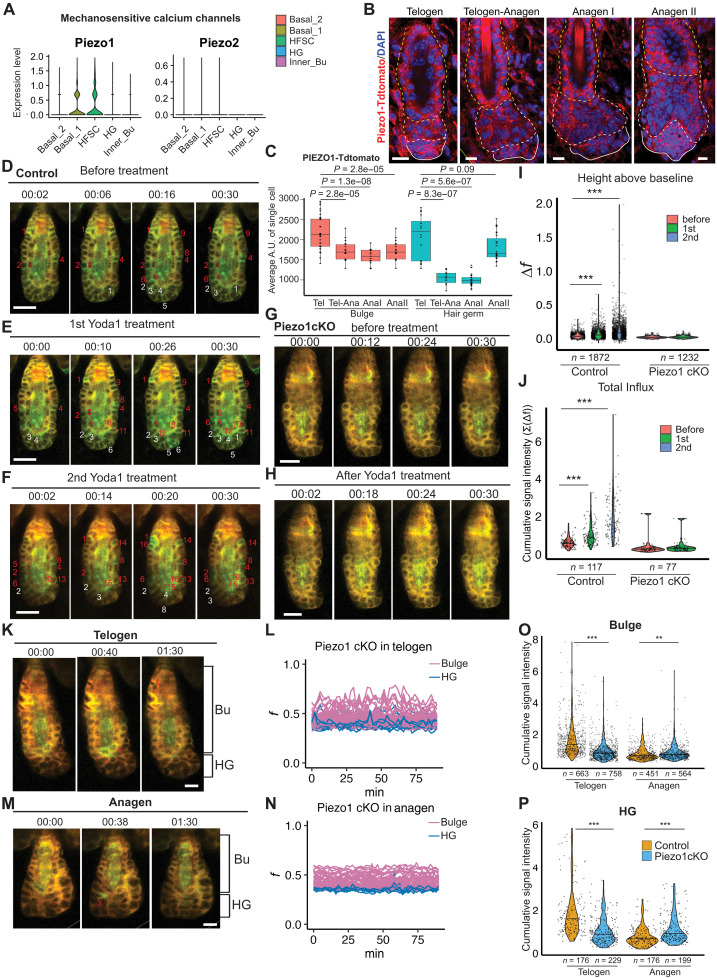
PIEZO1 mediates calcium influx in HF-SC compartments. (**A**) Expression pattern of *Piezo1* and *Piezo2* detected by scRNA-seq. (**B**) Immunofluorescence staining reveals PIEZO1 expression in bulge HF-SCs and HG cells during the first telogen and early anagen phases. Scale bars, 20 μm. (**C**) Quantification of Piezo1-tdT signals in (B) in the bulge and HG. *P* values are calculated by Student’s *t* test. (**D** to **F**) Yoda1 treatment (100 μl of 100 μM) increases calcium influx in control HFs. Time-lapse images show the progression of the calcium spikes. Red numbers indicate bulge cells with calcium influx events, and white numbers show HG cells with calcium influx events. Scale bars, 20 μm. (**G** and **H**) Yoda1 treatment does not increase calcium influx in *Piezo1* cKO HFs. Scale bars, 15 μm. (**I**) Quantification of calcium changes of control (left) and *Piezo1* cKO (right) HFs after Yoda1 treatment. *n* indicates the number of signal events from 4 pairs of animals, before and after Yoda1 treatment. Statistical significance was determined by Mann-Whitney U Test (***P < 0.001). (**J**) Cumulative calcium signals of control (left) and *Piezo1* cKO (right) after Yoda1 treatment. Statistical significance was determined by Mann-Whitney *U* test (***P < 0.001). (**K** and **M**) HF-SC compartments in *Piezo1* KO show reduced calcium dynamics in telogen (K) and early anagen (M). Scale bars, 10 μm. (**L** and **N**) Normalized calcium signal in *Piezo1* cKO bulge and HG cells over 90 min in telogen (L) and early anagen (N). (**O** and **P**) Deletion of *Piezo1* reduced cumulative calcium influx in HF-SC compartments including the bulge (O) and HG (P) in telogen. *n* indicates the number of cells from five control animals and six *Piezo1* cKO animals. Statistical significance was determined by Mann-Whitney *U* test (**P < 0.01 and ***P < 0.001). A.U., arbitrary unit.

To test this hypothesis, we investigated the molecular basis underlying the dynamic Ca^2+^ spiking patterns in bulge HF-SCs. We first examined recently published single-cell RNA sequencing (scRNA-seq) data, obtained from telogen skin at P53 (*14*), to determine the expression patterns of all Ca^2+^ channels, including voltage-gated Ca^2+^ channels, ligand-gated Ca^2+^ channels, transient receptor potential cation channels, and MS Ca^2+^ channels. In addition, we also examined the expression patterns of P2Y purinoceptor genes, which sense extracellular adenosine and uridine nucleotides and trigger Ca^2+^ release from the ER. Only *Piezo1* and *Trpv4* showed enriched expression in bulge HF-SCs among Ca^2+^ channels (Fig. 2A and fig. S5, A to C). Among P2Y receptors, only *P2ry1* showed low expression in basal interfollicular epidermis (IFE) populations (basal IFE 1 and 2), and all P2Y receptors were largely absent from HF cells (fig. S5D). Because PIEZO1 is an MS cation channel (*20*, *24*), we further examined the expression patterns of PIEZO1 in HF-SC and HG in telogen and early anagen by using a *Piezo1-tdTomato* knockin model (*25*). Consistent with the differential expression detected by scRNA-seq, PIEZO1 is strongly expressed in bulge HF-SCs in telogen. Notably, PIEZO1 expression is reduced in HGs during the telogen-to-anagen transition and in early anagen phases (Fig. 2, B and C).

Next, we determined the extent to which PIEZO1 governs Ca^2+^ influx in bulge HF-SC and HG progenitors. To this end, we recorded Ca^2+^ signals for 30 min and then applied Yoda1, a PIEZO1 agonist (*26*), to the skin of control (*Krt14-Cre/LSL-Salsa6f*) mice and recorded the Ca^2+^ signals again. For control mice, we applied Yoda1 twice sequentially and measured Ca^2+^ influx after each treatment for 30 min (Fig. 2, D to F, and movies S4). Quantification of the normalized Ca^2+^ signal changes (Δf) and the cumulative Ca^2+^ signals showed increasing Ca^2+^ influx after each treatment (Fig. 2, I and J, left panels). These results also suggest that homeostatic Ca^2+^ dynamics in the HF-SC compartment are at the similar scale of PIEZO1-mediated Ca^2+^ influx. To examine whether HF-SC Ca^2+^ dynamics is indeed mediated by PIEZO1, we generated a *Krt14-Cre/Piezo1^fl/fl^/LSL-Salsa6f* model, which ablated *Piezo1*, and measured Ca^2+^ signals in these mice. In contrast to control, *Piezo1* cKO skin did not respond to Yoda1 treatment (Fig. 2, G to J, and movie S5). We also noticed that *Piezo1* ablation appeared to significantly reduce Ca^2+^ influx, measured by both normalized Ca^2+^ signal changes (Δf) and the cumulative Ca^2+^ signals regardless of Yoda1 treatment (Fig. 2, I and J).

These observations prompted us to further monitored Ca^2+^ signals in bulge HF-SCs and HG progenitors of *Piezo1* cKO skin in telogen and early anagen. The Ca^2+^ signal intensity and cumulative Ca^2+^ signals in both telogen and early anagen were significantly reduced upon genetic deletion of *Piezo1* (Fig. 2, K to N). Quantification of cumulative Ca^2+^ influx in individual cells of both the bulge and HG revealed substantial reduction of Ca^2+^ levels in telogen (Fig. 2, O and P), reflecting a stronger impact of *Piezo1* deletion on Ca^2+^ influx during the quiescent telogen phase. We also noted that, however, Ca^2+^ influx in anagen showed *Piezo1-*independent changes, likely due to the relatively weak signal levels that approached the detection limit of our system. It is also possible that other channels, such as TRPV4, may function to regulate Ca^2+^ influx in anagen. The high-intensity Ca^2+^ spikes were not significantly affected by the loss of PIEZO1 (fig. S5E), indicating their independence of this channel. Together, these quantitative analyses suggest that PIEZO1 is the major Ca^2+^ channel in bulge HF-SCs in telogen.

### Force–through–E-cadherin triggers PIEZO1-mediated calcium flicker

**Fig. 3.**
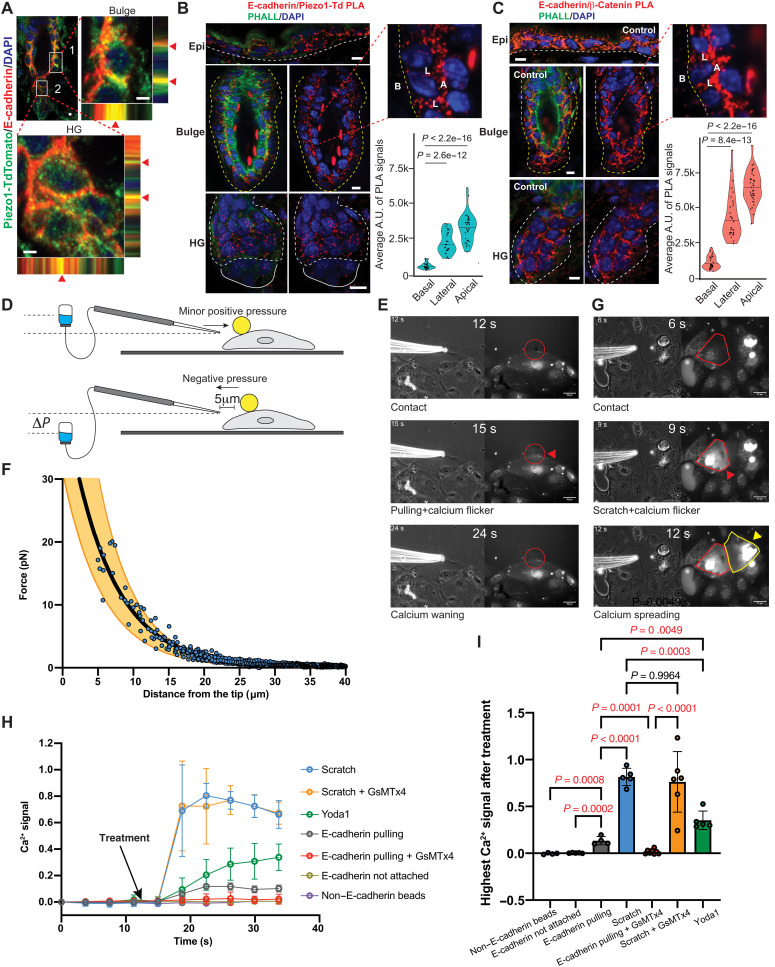
Force–through–E-cadherin triggers PIEZO1-mediated calcium flicker. (**A**) Colocalization of Piezo1-tdTomato and E-cadherin in bulge HF-SC and HG. Scale bars, 2 μm. (**B**) PLA shows the colocalization of Piezo1-tdTomato and E-cadherin. The insert shows the PLA signals in the lateral (L) and apical (A) sides but not the basal (B) side of the HF-SCs. *P* values are calculated by Student’s *t* test. Scale bars, 5 μm. (**C**) Colocalization of E-cadherin and β-catenin. The insert shows the PLA signals in the lateral (L) and apical (A) sides but not the basal (B) side of the HF-SCs. *P* values are calculated by Student’s *t* test. Scale bars, 5 μm. (**D**) Schematic illustration of the manipulation of E-cadherin–coated microbeads by the micropipette system. (**E**) Representative images of microbead manipulation (left, bright field) and calcium imaging (right, fluorescence) experiments. At 12 s, the micropipette contacts the microbead. At 15 s, the microbead is pulled by the micropipette, and a localized calcium flicker is recorded (red circle and arrowhead). At 24 s, the calcium signal is waned. (**F**) Force-distance curve is calibrated to determine the force (~20 pN) applied to the microbeads. The black and orange curves represent the averaged fit and range of 10 experiments, respectively. (**G**) Representative images of surface scratch (left, bright field) and calcium imaging (right, fluorescence) experiments. At 6 s, the micropipette contacts the cell surface, and no calcium flicker is recorded (red polygon). At 9 s, surface scratch activates strong calcium signal on the scratched cell (red polygon and arrowhead). At 12 s, calcium signal is spread to the neighboring cell (yellow polygon and arrowhead). (**H** and **I**) Quantification of calcium signals in the pulling and scratching experiments with or without Yoda1, a PIEZO1 agonist, and GsMTx4, a PIEZO1 inhibitor. Statistical significance is determined by ordinary one-way analysis of variance (ANOVA) test. DAPI, 4′,6-diamidino-2-phenylindole.

We next investigated how PIEZO1 senses mechanical forces in HF-SCs. While it is widely recognized that PIEZO1 is activated through the “force-from-lipids” mechanism (*27*), recent studies have shown that PIEZO1 directly interacts with E-cadherin in canine Madin-Darby canine kidney and human 293 cells (*28*). To determine whether PIEZO1 is tethered to the actin cytoskeleton through the adherens junction (AJ) in bulge HF-SCs, we performed immunostaining (IF) staining of PIEZO1-tdT together with E-cadherin. We observed that colocalized PIEZO1 and E-cadherin foci were highly abundant on the lateral side between bulge HF-SCs and on the apical side between bulge HF-SCs and inner bulge cells (Fig. 3A). To confirm the interaction between PIEZO1 and E-cadherin, we performed the proximity ligation assay (PLA) and detected colocalized PIEZO1 and E-cadherin in bulge HF-SCs and HG (Fig. 3B). As expected, colocalized PIEZO1 and E-cadherin signals were enriched in the interfaces of cell-cell interactions, including the lateral and apical side, but largely absent from the basal side, where cellbasement membrane interactions take place (Fig. 3B). Notably, colocalized PIEZO1 and E-cadherin foci were more abundant in bulge HF-SCs than HG progenitors (Fig. 3B). The specificity of the PLA was further confirmed by the positive interactions between E-cadherin and β-catenin (Fig. 3C) and the negative results from *Pizeo1* knockout (KO) (fig. S6A). The PLA signals were weaker for PIEZO1/E-cadherin than those of β-catenin/E-cadherin, suggesting a subset of E-cadherin/AJ interacting with PIEZO1. Collectively, these results reveal extensive colocalization of PIEZO1 and E-cadherin in cell-cell interaction interfaces in bulge HF-SCs.

We next tested whether PIEZO1 could sense mechanical forces through the interaction with E-cadherin and generate Ca^2+^ influx. To this end, we leveraged our E-cadherin microbead adhesion system (*14*, *29*), in which E-cadherin–coated microbeads (6.25 μm in diameter; fig. S6B) form homophilic bonds with E-cadherin on the surface of cultured primary keratinocytes. Instead of applying a strong force (3 to 5 nN) to break E-cadherin and actin linkage as originally designed (*14*, *30*), we reasoned that we could generate a force through E-cadherin by gently pulling the E-cadherin–attached microbead and monitor whether the mechanical force through E-cadherin activates PIEZO1 and triggers Ca^2+^ influx. To assure that the beads formed homophilic E-cadherin adhesion with the cells, we first applied a minor positive pressure to dislodge unattached beads away from the cell surface. This operation did not trigger any Ca^2+^ signals as indicated by the Fluo-4 Direct Ca^2+^ dye (movie S6), suggesting that unattached microbeads do not activate PIEZO1. In addition, microbeads without E-cadherin coating also did not trigger Ca^2+^ signals (quantified in Fig. 3, H and I). For microbeads not dislodged by the positive pressure, liquid aspiration was applied to capture those microbeads attached to the cells via E-cadherin. Because forces in the piconewton range are physiologically relevant for force transmission on individual linkages through integrin (*31*) and AJ (*32*), we aimed to generate the force at the pN scale. This was achieved by applying negative pressure through a hollow micropipette positioned approximately 5 μm from the microbead (Fig. 3D). This action triggered a weak and localized Ca^2+^ flicker underneath the displaced microbead (Fig. 3E and movie S7). The presence of GsMTx4, a PIEZO1 inhibitor, abolished these Ca^2+^ flickers, demonstrating the requirement of PIEZO1 (Fig. 3, H and I, and movie S8). Conversely, exposure to Yoda1, the PIEZO1 agonist, resulted in Ca^2+^ flickers that were about two to three times stronger than those elicited by pulling on the E-cadherin–attached microbeads (Fig. 3, H and I), a fold change that was similar to the responses observed in bulge HF-SCs in vivo.

Next, we quantified the forces generated during microbead manipulation that activated PIEZO1. A force of ~20 pN (Fig. 3F) was calculated based on the velocity (*v* = 261.2 ± 149.0 μm/s; fig. S6C) of the microbead moving toward the micropipette tip using the Stokes equation F=6πηrv, where $\eta =10^{-3}$ Pa·s and *r* = 3 μm represent the viscosity of the medium (fig. S6D) and the radius of the microbead, respectively. These findings demonstrate that a force of ~20 pN through E-cadherin is sufficient to activate PIEZO1, triggering weak and localized Ca^2+^ flickers in primary keratinocytes. Notably, this activation threshold is three orders of magnitude lower than the force required to activate PIEZO1 (~33 nN) by pulling the cell membrane through an ECM-coated bead (*33*), highlighting the sensitivity of PIEZO1 to the force through E-cadherin.

Because we observed strong Ca^2+^ spikes frequently in the epidermis and occasionally in HFs, we next examined how to trigger strong Ca^2+^ spikes and whether they are dependent on PIEZO1. Instead of gently pulling E-cadherin–attached microbeads, we used the micropipette to scratch the surface of cultured primary keratinocytes. Notably, surface scratch triggered strong Ca^2+^ spikes (Fig. 3G and movie S9) that are not only insensitive to PIEZO1 inhibitor but also rapidly spreading to neighboring cells (Fig. 3, G to I, and movie S10), recapitulating many features of strong Ca^2+^ spikes observed in the epidermis. Together, these results provide experimental evidence for PIEZO1 activation by the force through E-cadherin and define the nature of PIEZO1-mediated Ca^2+^ flicker as relatively weak and localized, distinct from strong and spreading Ca^2+^ spikes observed in the epidermis.

### PIEZO1 maintains quiescence of bulge HF-SCs

**Fig. 4.**
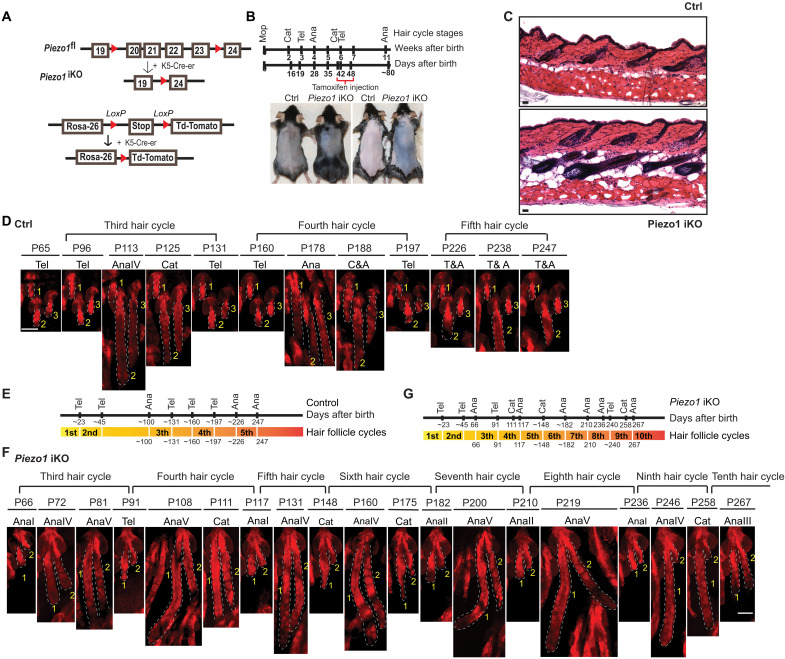
Deletion of *Piezo1* shortens the telogen length and promotes hair regeneration. (**A**) Schematics of the *Krt5-CreER*/*Piezo1*^fl/fl^ iKO model and a Rosa-LSL- tdTomato model for deleting *Piezo1* and marking the KO cells. (**B**) *Piezo1* iKO promotes dorsal hair regeneration upon the deletion in early telogen by administration of Tamoxifen from P42 to P48. Seven pairs of animals, four female and three male pairs, were used for phenotypical analysis. (**C**) H&E staining shows accelerated hair regeneration in *Piezo1* iKO. Scale bars, 100 μm. (**D**) Intravital imaging tracking of the same control HFs captures three hair cycles from P65 (the third hair cycle) to P247 (the fifth hair cycle). Forty HFs from two control animals were tracked for the study. Scale bar, 100 μm. (**E**) Timeline of hair cycle stages for control HFs tracked by intravital microscopy. (**F**) Intravital imaging tracking of the same *Piezo1* iKO HFs captures eight hair follicles from P66 (the third hair cycle) to P267 (the 10th hair cycle). Forty HFs from two iKO animals were tracked for the study. Note the progressively shortened telogen in the later cycles. Scale bar, 100 μm. (**G**) Timeline of hair cycle stages for *Piezo1* iKO HFs tracked by intravital microscopy.

Having characterized PIEZO1-mediated Ca^2+^ flicker in bulge HF-SCs, we next determined whether PIEZO1 plays a role in HF-SC functions. We generated a *Krt5-CreER/Piezo1^fl/fl^* inducible conditional KO [inducible KO (iKO)] model to delete *Piezo1* at the beginning of telogen (P42 to P48) (Fig. 4, A and B). Induced *Piezo1* deletion accelerated hair coat regeneration when control HFs still rested in telogen by P80 (Fig. 4, B and C). To monitor the hair cycle progression of individual HFs more precisely over time, we bred the iKO model to a *Rosa-LSL-tdTomato* reporter such that *Piezo1-*deleted HFs were visualized by tdT signals. We tracked the same HFs in control and *Piezo1* iKO mice for their hair cycle between P45 and P267. During the 7-month window, control HFs regenerated three times with each telogen phase lasting 30 to 50 days (Fig. 4, D and E), consistent with previous findings (*34*, *35*). In contrast, during the same period, *Piezo1* iKO HFs regenerated eight times (from cycle #3 to cycle #10), with each telogen phase lasting ~10 to 21 days (Fig. 4, F and G). Notably, while *Piezo1* KO HF-SCs still enter the quiescent telogen, the duration of telogen progressively decreased from ~21 days after the initial deletion of *Piezo1* (cycle #3) to ~10 days by the 10th hair cycle (Fig. 4F), suggesting an accumulative effect of *Piezo1* ablation on HF-SC quiescence. In addition, the *Krt-14/Piezo1^fl/fl^* cKO model recapitulated the phenotype of shortened telogen and more frequent HF regeneration (fig. S7, A to D). These longitudinal data demonstrate that ablation of *Piezo1* increased the frequency of HF growth with compromised HF-SC quiescence.

### Deletion of *Piezo1* compromises mechanical properties of quiescent HF-SCs

**Fig. 5.**
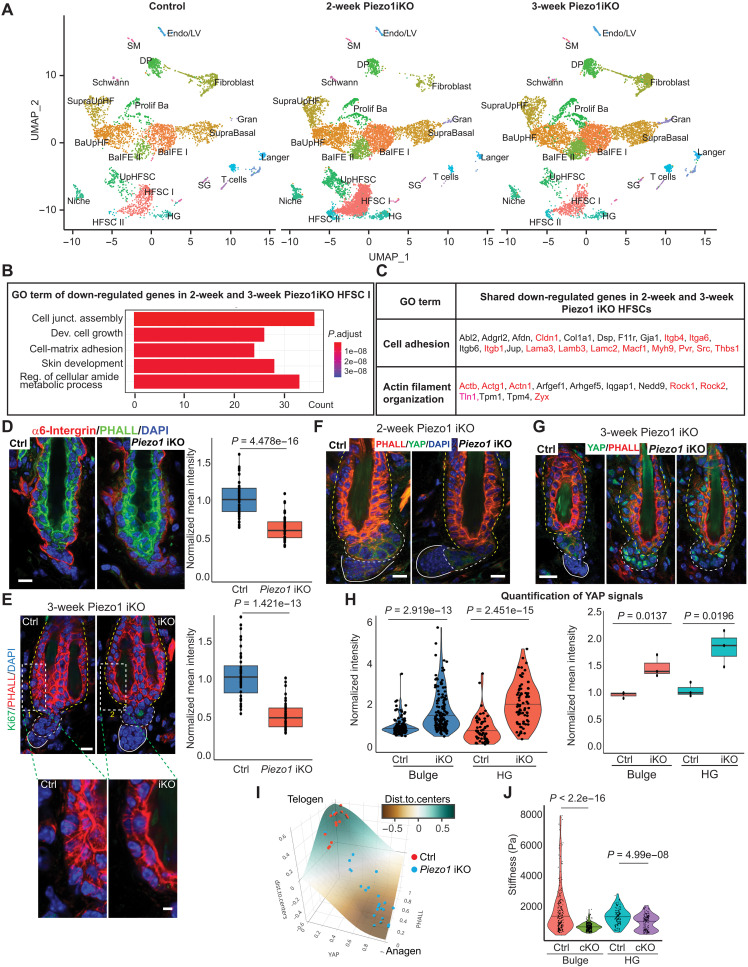
Deletion of *Piezo1* compromises mechanical properties of quiescent HF-SCs. (**A**) Time series of scRNA-seq datasets from control and *Piezo1* iKO samples. (**B**) Top five categories of Gene Ontology (GO) terms of down-regulated genes shared in 2- and 3-week *Piezo1* iKO HF-SCs compared to the control. (**C**) Shared down-regulated genes in 2- and 3-week *Piezo1* iKO HF-SCs are enriched in cell adhesion and actin filament organization categories. Red labeled genes indicate notable genes in these functions. (**D**) α6-Intergrin levels in bulge and HG are decreased in *Piezo1* iKO 3 weeks after the deletion (ctrl, *n* = 51 cells; iKO, *n* = 45 cells; three pairs of animals). Scale bar, 10 μm. (**E**) Phalloidin staining reveals reduced stress fiber formation in bulge HF-SCs upon *Piezo1* deletion (ctrl, *n* = 40 fibers; iKO, *n* = 46 fibers; three pairs of animals). Scale bars, 10 μm (top) and 5 μm (insert). (**F**) Nuclear YAP1 is not accumulated in *Piezo1* iKO HFs 2 weeks after the deletion. Scale bars, 10 μm. (**G**) Increased nuclear YAP1 accumulation is observed in bulge HF-SCs and HGs 3 weeks after the deletion. Scale bar, 10 μm. (**H**) Quantification of YAP signals: 118 HF-SCs and 60 HG cells from three ctrl; 135 HF-SCs and 77 HG cells from three iKO. *P* values are calculated by Student’s *t* test for (D), (E), and (H). (**I**) *Piezo1* iKO promotes the transition from the quiescent telogen phase to the activated anagen phase, determined by F-actin and nuclear YAP1 signals and modeling. (**J**) Stiffness of the bulge and HG in dorsal skin, measured by AFM, is reduced upon depletion of *Piezo1*. Ctrl, *n* = 4 HFs; iKO, *n* = 5 HFs. Ctrl: bulge mean = 1965.4 ± 736.0 Pa, HG mean = 1371.8 ± 491.6 Pa. Piezo1 iKO: bulge mean = 719.0 ± 239.3 Pa, HG mean = 1002.2 ± 491.6 Pa. *P* value is determined by Mann-Whitney *U* test.

Deletion of *Piezo1* impairs Ca^2+^ influx and shortens the quiescent telogen phase. To probe the mechanism through which Ca^2+^ signaling governs HF-SC activities, we performed a time series of scRNA-seq on skin samples obtained 2 and 3 weeks following induced deletion of *Piezo1*, a period during which control and *Piezo1* iKO HFs remain in telogen (fig. S8A). Analysis of epithelial and nonepithelial cell populations from control and both iKO samples revealed similar percentages and clustering patterns, suggesting relatively minor perturbation in cell state due to *Piezo1* deletion (Fig. 5A and fig. S8, B and C). Based on well-established lineage markers (fig. S8D), we resolved upper HF-SC, one major HF-SC population, one minor HF-SC population, the inner bulge niche cells, and an HG population (Fig. 5A and fig. S8, B to D), consistent with previous studies from telogen HFs (*36*–*38*). While *Piezo1* ablation causes differential gene expression in interfollicular epidermis and HF-SCs, we focused on the effect on HF-SCs. In the HF-SC cluster, the 2-week and 3-week samples shared ~50% of down-regulated genes, and the 3-week sample had more down-regulated genes (33.8%) than the 2-week sample (16.2%), reflecting progressive changes of the transcriptome upon *Piezo1* deletion (fig. S8E).

Genes associated with cell adhesion, the ECM, and the actin cytoskeleton—including *Actg1, Actn1*, *Itgb1*, *Itgb4*, *Itga6*, *Laminin-332* (*Lama3, Lamb3, Lamc2*)*, Macf1*, *Rock1, Rock2*, *Thbs1, Tln1* and *Zyx*—were among the most highly enriched in the commonly down-regulated genes in both the 2- and 3-week samples (Fig. 5, B and C, and data S1). Nearly all of these genes were more strongly down-regulated in the 3-week sample compared to the 2-week counterparts, reflecting progressive reduction of these cell adhesion and actin cytoskeleton genes upon *Piezo1* ablation. Genes associated with HF-SC activation and re-entry into the cell cycle—including *Cenpe*, *Cenpf*, *mKi67* and *Top2a* (*35*, *39*)—did not show significantly elevated expression in HF-SCs from the 2-week or 3-week time points (fig. S8F). The S and G_2_-M scores, which take into consideration all cell cycle–related genes, did not show differences across all three samples at this stage (fig. S8G). Genes associated with translation were notably up-regulated (data S2), likely reflecting increased protein synthesis that is required for HF-SC activation (*40*). These data suggest that changes in ECM, actin cytoskeleton, and cell adhesion are downstream of *Piezo1* deletion before the cell cycle re-entry.

IF staining of α6-integrin confirmed down-regulation in bulge HF-SCs and HG progenitors but not in interfollicular epidermis (Fig. 5D and fig. S9A). Consistent with impaired gene expression in focal adhesion and actin cytoskeleton, stress fiber formation, a feature of bulge HF-SCs (*14*), was reduced in *Piezo1* iKO (Fig. 5E), reflecting decreased cell adhesion. We next examined the dynamics of nuclear yes-associated protein 1 (YAP1) accumulation, which is sensitive to mechanical cues and drives cell cycle re-entry in epithelial cells of the skin (*41*–*43*). Two weeks following the induced deletion of *Piezo1*, YAP1 had not yet localized to the nuclei of HF-SCs and HG progenitors (Fig. 5F). However, by the third week post-deletion, there was a noticeable increase in nuclear YAP1 accumulation in both compartments (Fig. 5G). The reduced stress fiber formation and nuclear localization of YAP1 in both HF-SCs and HG progenitors in *Piezo1* iKO skin suggests perturbed force generation and mechanical sensing in these compartments upon the loss of *Piezo1.* It was recently demonstrated that quantitative measurement of F-actin signal intensity and nuclear YAP1 can distinguish the transition from quiescent telogen to active anagen phase (*14*). Plotting of F-actin and nuclear YAP1 signals in *Piezo1* iKO HF-SCs revealed their cell state dynamically transitioning from telogen toward anagen (Fig. 5H and see Materials and Methods) before the cell cycle re-entry as indicated by the transcriptomic analysis (fig. S8, F and G). To determine whether compromised mechanical sensing in *Piezo1* KO disrupts the mechanical properties of bulge HF-SCs, we applied AFM to measure the stiffness of the bulge and HG compartments, respectively. The composite stiffness of the bulge was significantly reduced from an average of 1965.4 to 719.0 Pa, whereas HG showed a milder but still significant reduction from 1371.8 to 1002.2 Pa (Fig. 5I). Together, these data reveal a requirement of PIEZO1 in maintaining mechanical properties, including tissue stiffness and robust actin cytoskeleton, of the bulge and HG.

### PIEZO1 regulates a transcriptional network in quiescent HF-SCs

**Fig. 6.**
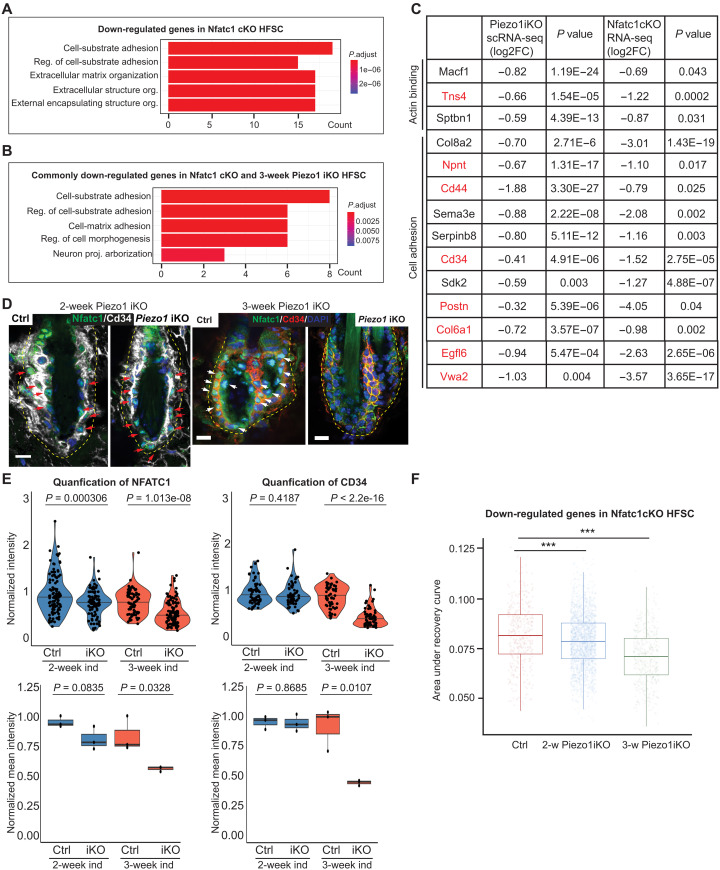
PIEZO1 controls gene expression through transcription factor NFATC1. (**A**) Top five GO terms of down-regulated genes in *Nfatc1* cKO HF-SCs bulk RNA-seq data. (**B**) GO term analysis of commonly down-regulated genes in *Nfatc1* cKO and *Piezo1* iKO HF-SCs. (**C**) Gene lists of down-regulated NFATC1 targets involved in cell adhesion and actomyosin network in 3-week *Piezo1* iKO HF-SCs. Red colored genes are only down-regulated in the 3-week sample but not in the 2-week sample. (**D**) Deletion of *Piezo1* reduces NFATC1 nuclear localization in HF-SCs after 3 weeks (right) but not 2 weeks (left) after the induced deletion. Scale bar, 10 μm. (**E**) Quantification of NFATC1 and CD34 in bulge HF-SCs in control and *Piezo1* iKO after 2- and 3-week treatment. A total of 113 bulge HF-SCs from three control mice and 85 bulge HF-SCs from 3 *Piezo1* iKO mice after 2-week treatment and 74 bulge HF-SCs from three control mice and 122 bulge HF-SCs from 3 *Piezo1* iKO mice after 3-week treatment are quantified for nuclear NFATC1. A total of 58 bulge HF-SCs from three control mice and 59 bulge HF-SCs from three *Piezo1* iKO mice after 2-week treatment and 54 bulge HF-SCs from three control mice and 64 bulge HF-SCs from three *Piezo1* iKO mice after 3-week treatment are quantified for CD34. Each dot in the violin plot represents normalized value in each cell for nuclear NFATC1 or CD34, respectively. Dots in box plot show normalized mean intensity of nuclear NFATC1 or CD34 from each animal, respectively. *P* values are calculated by Student’s *t* test. (**F**) NFATC1-dependent genes are progressively down-regulated from 2 to 3 weeks after deletion in *Piezo1* iKO HF-SCs. Statistical significance was determined by Wilcoxon rank sum test (***P < 0.001).

We next investigated how PIEZO1-mediated Ca^2+^ signals regulate the expression of ECM, cell adhesion, and actin cytoskeleton genes. The widespread impact on cell adhesion and actin cytoskeleton genes caused by *Piezo1* deletion is reminiscent of the perturbed transcriptome detected in *Nfatc1* cKO HF-SCs (Fig. 6, A and B) (*35*)*,* a key TF known for its role in regulating HF-SC quiescence and aging (*35*, *44*, *45*). Notably, many validated NFATC1 targets in HF-SCs—such as *Cd34*, *Npnt*, and *Col6a1*—were down-regulated in *Piezo1* iKO HF-SCs 3 weeks after ablation (Fig. 6C, fig. S9B, and data S3). The mRNA levels of HF-SC TFs—including *Nfatc1*, *Sox9*, and *Lhx2* (*44*, *46*–*50*)—remained unchanged (fig. S9C). We further examined the expression of NFATC1 in control and *Piezo1* iKO skin with IF staining. While NFATC1 was still localized to the nuclei of HF-SCs 2 weeks after *Piezo1* deletion, these nuclear signals were largely depleted, accompanied by CD34 reduction, by 3 weeks after the induced deletion (Fig. 6, D and E). The reduced NFATC1 nuclear accumulation was consistent with the observation that genes down-regulated upon *Nfatc1* cKO were also down-regulated in *Piezo1* iKO HF-SCs. Furthermore, many of these NFATC1 targets were only down-regulated in the 3-week sample but not in the 2-week sample (Fig. 6C and data S4 and S5), mirroring the nuclear depletion of NFATC1. To further examine the dynamics of NFATC1-regulated transcriptome in *Piezo1* iKO samples, we leveraged the scRNA-seq datasets obtained following 2- and 3-week *Piezo1* deletion and examined the dynamics of NFATC1*-*dependent genes. We detected progressive down-regulation of NFATC1*-*dependent genes following the induced deletion of *Piezo1* (Fig. 6F). These data suggest that NFATC1 nuclear localization and function are dependent on PIEZO1-mediated Ca^2+^ signaling for the regulation of HF-SC quiescence.

**Fig. 7.**
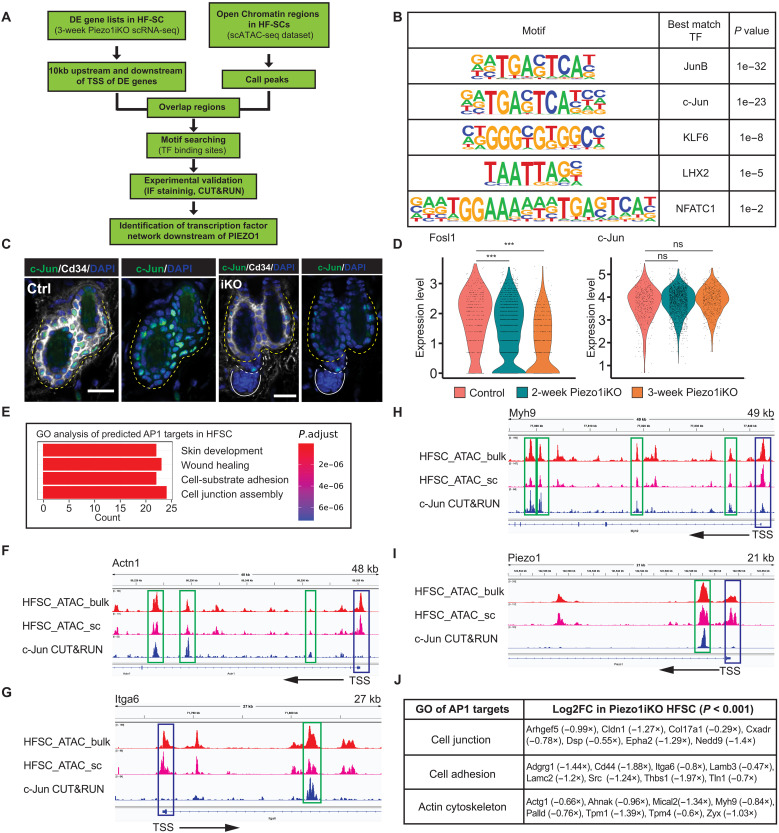
PIEZO1 regulates the expression of cell adhesion and actin genes through AP1 transcription factors. (**A**) Schematics for identifying transcription factors downstream of PIEZO1 by using scRNA-seq and scATAC-seq datasets. (**B**) Highly enriched transcription factor motifs within ±10 kb of the TSS of down-regulated genes in *Piezo1* KO HF-SCs. (**C**) c-Jun nuclear localization is reduced in the bulge, marked by CD34, after 3-week induction of *Piezo1* deletion. Scale bars, 20 μm. (**D**) mRNA levels of *Fosl1* but not *c-Jun* are progressively decreased in HF-SCs in *Piezo1* iKO as determined by scRNA-seq (***P < 0.001, ns, not significant). (**E**) Top four GO terms of predicted AP1 targets in HF-SCs. (**F** to **I**) IGV track of HF-SC bulk ATAC-seq, HF-SC scATAC-seq, and AP1 CUT&RUN data in the loci of *Actn1* (F), *Itga6* (G), *Myh9* (H) and *Piezo1* (I). Blue box indicates the TSS, green box indicates the c-Jun binding, and arrow indicates the direction of the transcription. (**J**) Representative gene lists of down-regulated AP1 targets that are highly enriched in cell junction, adhesion, and actin cytoskeleton categories in *Piezo1* iKO HF-SCs after 3 weeks of induced deletion.

We next globally identified transcription factors, which function downstream of PIEZO1-dependent Ca^2+^ signaling. We used previously established single-cell Assay for Transposase Accessible Chromatin (ATAC) sequencing (scATAC-seq) data (*35*) and identified open chromatin landscape across all major skin cell populations including HF-SCs (fig. S9D). We then searched for the most highly enriched TF motifs within a ±10-kb region of the transcription start site (TSS) of down-regulated genes detected in *Piezo1* iKO HF-SCs (Fig. 7A). AP1, KLF6, LHX2, and NFATC1 motifs were detected as highly enriched (Fig. 7B). While the identification of LHX2 and NFATC1 motifs provided further evidence for the regulation of HF-SC quiescence through PIEZO1, the identification of AP1 and KLF6 as the most highly enriched TF motifs in response to PIEZO1-mediated sensing of mechanical cues was intriguing. We first turned to c-Jun (also known as JUN), whose nuclear localization and transcriptional activity are controlled by JNK (c-Jun N-terminal kinase) in a Ca^2+^-dependent manner (*51*). Nuclear-localized c-Jun was significantly reduced in *Piezo1* iKO bulge (Fig. 7C and fig. S9E), consistent with the reduced Ca^2+^ dynamics in *Piezo1* null HF-SCs. Similar to *Nfatc1*, *c-Jun* mRNA levels were not perturbed in *Piezo1* iKO. In contrast, mRNA of *Fosl1*, another member of the AP1 TF family, was significantly reduced in bulge HF-SCs upon *Piezo1* deletion (Fig. 7D). Closer inspection of predicted AP1 targets revealed high enrichment in the categories of skin development, cell-substrate adhesion, and cell junction assembly (Fig. 7E and data S6). While AP1 TFs are known to regulate cell proliferation, epidermal differentiation, stress response, and tumorigenesis (*52*–*55*), their functions in bulge HF-SCs remain largely unclear at least, in part, due to the lack of understanding of their regulated genes in these quiescent SCs. To probe how AP1 TFs regulate gene expression in the bulge, we performed c-Jun CUT&RUN in fluorescence-activated cell sorting (FACS) purified HF-SCs (K14-H2bGFP^hi^/Sca1^neg^/CD34^hi^/CD49f^hi^). From the sequencing results, we detected strong signal-to-noise ratio and the robust enrichment of AP1 TF motifs detected in the CUT&RUN peaks and observed the depleted reads in DNA regions harboring c-Jun motifs that are protected by the TF binding (fig. S10, A and B). These results validated technical performance of c-Jun CUT&RUN. We confirmed that most of the predicted AP1 targets (263 of 326 genes, 81%) are indeed bound by c-Jun in their promoters or proximal enhancers. Many functionally important targets involved in cell adhesion, ECM, and actin cytoskeleton—such as *Actg1*, *Actn1*, *Itga6*, *Myh9*, *Rock2*, *Thbs1*, *Zyx*—were confirmed to harbor strong c-Jun CUT&RUN signals (Fig. 7, F to H and fig. S10C). We also detected strong binding of c-Jun to transcription factors, such as *Klf6*, *Stat3*, *Rela*, and post-transcriptional regulators, such as *miR-205* and the *miR-200b/a/429* cluster (fig. S10D). Intriguingly, the *Piezo1* locus also harbors a strong c-Jun binding site within the first intron, ~2.3 kb downstream of the TSS (Fig. 7I), suggesting a possibility that *Piezo1* expression is also controlled by c-Jun/AP1 TFs in bulge HF-SCs. Overall, CUT&RUN-validated AP1 targets were most highly enriched in genes associated with cell junction, cell adhesion, and actin cytoskeleton (Fig. 7J). These data reveal that AP1 TFs regulate gene expression associated with mechanical properties of HF-SCs.

**Fig. 8.**
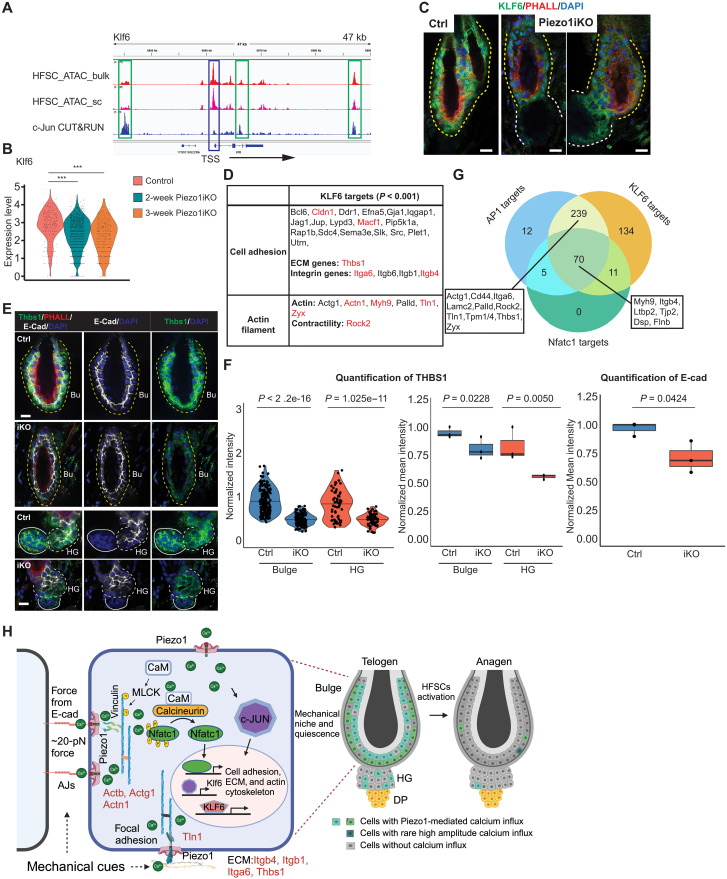
The transcriptional network downstream of PIEZO1-mediated calcium influx reinforcing quiescence and mechanical properties of HF-SCs. (**A**) IGV track of HF-SC bulk ATAC-seq, HF-SC scATAC-seq, and c-Jun CUT&RUN data in *Klf6* locus. Blue and green boxes indicate the TSS and c-Jun binding, respectively. An inactive noncoding RNA is located upstream of the TSS of *Klf6.* (**B**) mRNA levels of *Klf6* are progressively reduced in HF-SCs in 2-week and 3-week *Piezo1* iKO scRNA-seq datasets (***P < 0.001). (**C**) KLF6 levels are decreased in *Piezo1* iKO bulge. Scale bars, 10 μm. (**D**) Predicted KLF6 targets show significant enrichment in the categories of cell adhesion and actin filament. Red colored genes are also targeted by c-Jun/AP1. (**E**) THBS1 proteins are reduced in the bulge and HG 3 weeks after induced *Piezo1* deletion. Scale bars, 10 μm. (**F**) Quantification of THBS1 signals in bulge HF-SCs and HG cells in control and *Piezo1* iKO (left) and quantification of E-cadherin signals in bulge HF-SCs (right). A total of 178 bulge HF-SCs and 63 HG cells from three control mice and 145 bulge HF-SCs and 68 HG cells from 3 *Piezo1* iKO mice are quantified for THBS1. A total of 60 bulge HF-SCs from three control mice and 48 bulge HF-SCs from three *Piezo1* iKO mice are quantified for E-cadherin. Each dot in the violin plot represents normalized value for THBS1 in each cell. Dots in box plot show normalized mean intensity of THBS1 or E-cadherin from each animal. *P* values are calculated by Student’s *t* test. (**G**) Venn diagram of AP1, NFATC1, and KLF6 targets in *Piezo1* KO HF-SCs. (**H**) Model of PIEZO1-mediated calcium influx controlled by the force from E-cadherin reinforcing quiescence and mechanical properties of HF-SCs through the regulation of ECM, cell adhesion, and actin cytoskeleton by transcription factors AP1, NFATC1, and KLF6.

Because KLF6 was identified as a potential TF regulator downstream of PIEZO1 and the *Klf6* locus harbors multiple strong c-Jun enhancers (Fig. 8A), we further examined KLF6-mediated gene expression. *Klf6* was progressively down-regulated at mRNA levels in *Piezo1* iKO HF-SCs (Fig. 8B). Reduced KLF6 expression was confirmed by IF staining in *Piezo1* iKO HF-SCs (Fig. 8C), supporting the notion that *Klf6* is a target of c-Jun/AP1 TFs. Furthermore, KLF6 targets, defined by down-regulated genes harboring KLF6 binding motifs within 10 kb of their TSSs, were highly enriched for cell-matrix adhesion and the regulation of actin cytoskeleton (Fig. 8D). Among these targets, *Thbs1* (thrombospondin1) is prominent for its role in mechanotransduction (*56*), and its expression is required to maintain cell stiffness through the formation of stress fibers (*57*). IF staining confirmed strong down-regulation of Thrombospondin-1 (THBS1) in both bulge HF-SCs and HG progenitors (Fig. 8, E and F), consistent with the reduced stress fiber formation and reduced stiffness detected in both compartments. *Thbs1* is also targeted by c-Jun/AP1 (fig. S10C), representing many of these down-regulated genes cotargeted by the AP1/KLF6/NFATC1 network. Among 527 down-regulated genes upon *Piezo1* deletion, we have identified 70 genes (13.3%)—including *Itgb4*, *Myh9*, *Ltbp2*, *Tjp2*, coregulated by NFATC1, AP1, and KLF6 and 239 genes (45.4%)—including *Actg1*, *Palld*, *Rock2*, *Thbs1*, *Tln1*, and *Zyx*—coregulated by AP1 and KLF6 (Fig. 8G). In contrast, only 56 genes (10.6%) were not targeted by any of these TFs. Collectively, these studies identify a transcriptional network, consisting of AP1, KLF6, and NFATC1, functions downstream of PIEZO1 to reinforce the expression of genes associated with mechanical properties of HF-SCs.

## DISCUSSION

In this study, we have identified the low amplitude and localized Ca^2+^ flickers, mediated by PIEZO1, as a key mechanism by which HF-SCs sense the mechanical cues of their microenvironment. We propose a model in which PIEZO1 responds to the mechanical forces, exerted through E-cadherin at cell-cell junctions, facilitating a weak yet persistent Ca^2+^ influx in quiescent HF-SCs. In response to cumulative Ca^2+^ levels, the transcription factors NFATC1 and c-Jun translocate to the nucleus and maintain their localization there, promoting the expression of genes related to the ECM, actin cytoskeleton, and cell adhesion. This mechanism allows HF-SCs to translate mechanical information into biochemical signals, which in turn govern cellular activities. Thus, PIEZO1 is pivotal not only in sensing the mechanical properties of bulge HF-SCs but also in reinforcing their cellular state by controlling gene expression through a Ca^2+^-dependent TF network (Fig. 8H). In the absence of *Piezo1*, HF-SCs lose the ability to sense the stiff microenvironment and reinforce their quiescence through the NFATC1 and AP1 transcriptional network (fig. S10E). This dual role underscores the integral function of PIEZO1 in coordinating cellular responses to mechanical stimuli, essential for maintaining the structural integrity and functionality of HF-SC compartment. While this work was under review, a study in mouse intestinal SCs reported a requirement of *Piezo1* and *Piezo2* mediated mechanical sensing for both the maintenance and multipotency of these tissue SCs through the regulation of NOTCH and WNT signaling pathways (*58*). While deletion of *Piezo1* compromises HF-SC quiescence and the mechanical property of the niche, loss of *Piezo1/Piezo2* leads to enhanced ISC proliferation, resulting in the depletion of ISCs. Collectively, these results have unraveled the critical and versatile functions of PIEZO channels in mammalian tissue SCs.

### PIEZO1 mediates cumulative calcium influx in HF-SCs

While it is widely recognized that mechanical cues regulate SC functions, the specific mechanisms that link mechanical sensing to transcriptomic responses within tissues, such as the HF, are not yet fully understood. In the epidermis, Ca^2+^ signaling plays important roles in maintaining homeostasis and responding to wounds in an ATP-dependent manner (*8*, *15*, *22*, *23*). However, it is not clear whether Ca^2+^ influx directly contributes to mechanosensing in HF-SCs. Leveraging the distinct mechanical properties of bulge HF-SCs and HG progenitors and applying ratiometric Ca^2+^ imaging in intact tissues, we demonstrated that cumulative Ca^2+^ influx is correlated with changes in tissue stiffness and actomyosin contractility in these cells during quiescence and activation. Mechanical pulling on E-cadherin–coated microbeads, exerting a force of ~20 pN, activates PIEZO1, resulting in localized Ca^2+^ flickers. Previously, it has been shown that forces ranging from 5 to 30 pN are sufficient to unfold α-catenin and enhance α-catenin–vinculin interaction at AJs (*32*). Moreover, the activating force of ~20 pN through E-cadherin is three orders of magnitude lower than the pulling force of ~30 nN required through collagen IV (*33*) for PIEZO1 activation, highlighting the high sensitivity of PIEZO1 to the force through E-cadherin. Our findings thus provide key experimental evidence supporting the force-from-filament model of PIEZO1 mechanosensing (*28*) and identify AJs as critical mediators of PIEZO1-dependent Ca^2+^ influx in HF-SCs.

Unlike the transient and intense Ca^2+^ spikes observed in the epidermis, Ca^2+^ influx in these SCs is relatively weak yet persistent, likely reflecting the constant sensing by PIEZO1. While epidermal Ca^2+^ spikes frequently spread to neighboring cells, which are controlled by P2Y receptor–mediated ATP sensing (*22*) or coordinated through gap junctions (*15*), the Ca^2+^ influx detected in HF-SCs appears sporadic and remains localized within individual cells. These features could endow individual HF-SCs with the ability to respond to spatial differences in the mechanical properties of their microenvironment and contribute to the heterogeneity of cell states.

Analysis of Ca^2+^ channel expression patterns by scRNA-seq reveals that both *Piezo1* and *Trpv4* are enriched in HF-SCs. A previous study demonstrated a requirement of TRPV4 activation in promoting telogen to anagen transition through a Ca^2+^-dependent ERK pathway (*59*). Because no Ca^2+^ imaging was performed in *Trpv4* KO HF-SCs, it remains unclear about the extent to which Ca^2+^ influx is controlled by TRPV4 and the spatiotemporal patterns of TRPV4-mediated Ca^2+^ influx. A recent study demonstrated that Ca^2+^ influx mediated by TRPV6, but not PIEZO1, plays a key role in transendothelial migration (*60*), highlighting a possibility that spatiotemporal control of Ca^2+^ influx and their associated downstream events further dictates physiological responses to increased intracellular Ca^2+^. A previous report revealed a role of PIEZO1 and its downstream tumor necrosis factor–α in inducing HF-SC apoptosis during HF miniaturization and aging (*61*). The measurement of bulge stiffness indicates that aged bulge is more rigid than their young counterparts (*14*, *16*), which likely to induce more potent Ca^2+^ influx. These observations raise the possibility that different levels of Ca^2+^ influx mediated by PIEZO1 trigger different downstream responses in a context-dependent manner. It is possible that enhanced detection of Ca^2+^ signals in vivo would permit better mechanistic understanding of how individual HF-SCs sense and respond to their microenvironment in homeostasis, wound repair, and aging.

### Cumulative calcium influx regulates a transcription network in HF-SCs

Although transcription factor NFATC1 has been well characterized for its Ca^2+^-dependent nuclear localization in T cells (*62*, *63*), it remains unclear how the nuclear localization of NFATC1 is maintained in HF-SCs. Notably, NFATC1 localization in the bulge and its transcriptional activity are disrupted by cyclosporine A (*44*), consistent with the notion that NFATC1 is sensitive to cytosolic Ca^2+^ in the bulge. Furthermore, NFATC1 activation requires prolonged, rather than transient, Ca^2+^ spikes (*62*, *63*), a requirement that is matched by PIEZO1-dependent Ca^2+^ patterns observed in the bulge. Deletion of *Piezo1* leads to progressive depletion of NFATC1 from the nuclei, whereas mRNA levels remain constant. The time series of scRNA-seq performed 2 and 3 weeks after *Piezo1* deletion reveal a gradual decrease of NFATC1-regulated genes, lending support to PIEZO1-dependent NFATC1 function.

In addition to NFATC1, c-Jun is also depleted from the nuclei of PIEZO1 deleted HF-SCs while maintaining its mRNA levels. c-Jun/AP1 are versatile and enigmatic TFs, which have been implicated in numerous functions in wound healing, cell proliferation, epidermal differentiation, and tumorigenesis (*51*, *54*, *55*). However, their regulated genes in HF-SCs are highly enriched in ECM, cell adhesion, and actin cytoskeleton genes, which are important for the maintenance of mechanical property and quiescence of HF-SCs (Fig. 8H). These findings establish a molecular basis to examine the functions of AP1 TFs in homeostasis, stress response, and aging of HF-SCs. Furthermore, ~80% of down-regulated genes upon the loss of PIEZO1 can be explained by the regulation of NFATC1 and AP1 and their downstream target, KLF6. This result suggests that the AP1-NFATC1-KLF6 network is the major downstream effector of PIEZO1-mediated Ca^2+^ influx in HF-SCs. These studies of early events upon PIEZO1 depletion, however, did not examine the mechanisms that directly trigger HF-SC proliferation and differentiation. We note that, however, YAP1 nuclear localization was readily detected just before the cell cycle re-entry (Fig. 5, F to H). It is possible that YAP1 senses the mechanical changes, caused by the loss of PIEZO1, and drives the cell cycle re-entry.

### PIEZO1 reinforces HF-SC stiffness and quiescence

The mechanical architecture of the bulge HF-SC and HG progenitor compartments is notable such that the SC compartment is stiff and quiescent, whereas the progenitor compartment is relatively soft and primed for cell cycle re-entry (*14*). This is in contrast to the epidermis where the proliferative basal progenitors reside in a soft microenvironment, whereas differentiated cells, which exit the cell cycle, are more rigid (*6*). In intestinal organoids, increased substrate stiffness (>1.5 kPa) leads to a bulge-like morphology of the intestinal SC compartment (crypt), whereas soft substrates (~0.7 kPa) lead to crypt folding (*18*), similar to the morphology of the HG. Furthermore, the recent ISC study also revealed that the bottom of the crypt, the ISC niche, is more rigid than the top of the crypt (*58*). These data suggest that ECM and substrate stiffness controls the morphology and SC activities of epithelial tissues. Bulge HF-SCs express a specific set of ECM and cell adhesion genes (*64*), which are important for the integrity of the compartment and the quiescence of these cells (*35*). On the other hand, the pronounced stress fiber formation and high pMLC2 levels indicate strong focal adhesion formation and actomyosin contractility in bulge HF-SCs. Our findings demonstrate that PIEZO1-mediated transcriptional network control gene expression of both actin cytoskeleton—such as *Actg1*, *Actn1*, *Myh9*, *Rock1/2*, and *Tln1*—and ECM, such as *Igta6* and *Thbs1.* Upon *Piezo1* deletion, bulge HF-SCs progressively alter NFATC1 and c-Jun nuclear retention and their transcriptome and mechanical properties (fig. S10E), and it is not until 3 weeks after the initial deletion when HFs transition from the quiescent telogen phase to active anagen. This timeframe is comparable to the effect of PIEZO1-controlled SC differentiation in the adult *Drosophila* midgut (*65*). Collectively, these findings provide experimental evidence for the maintenance of HF-SC stiffness and quiescence through PIEZO1-mediated mechanosensing and cumulative Ca^2+^ influx.

## MATERIALS AND METHODS

### Animal studies

All mice breeding and operation procedures were approved by the Institutional Animal Care and Use Committees at Northwestern University Feinberg School of Medicine (IL, USA) and in accordance with the regulations and guidelines for the care and use of laboratory animals. Northwestern University has an Animal Welfare Assurance on file with the Office of Laboratory Animal Welfare (A3283-01). The *Piezo1* fl/fl (Jax, # 029213), *LSL-Salsa6f* (Jax, # 031968), *Piezo1-TdTamato* (Jax, #029214), and *Rosa26-LSL-tdTomato* (Jax, # 021876) mice were obtained from the Jackson Laboratory. *Piezo1* fl/fl mice were bred with *Krt14*-*Cre* (E. Fuchs, Rockefeller University) or *Krt5*-*CreER* (JAX # 018394) to generate *Piezo1* cKO or *Piezo1* iKO, respectively. *LSL-Salsa6f* mice were bred with *Krt14*-*Cre* to generate epithelial specific calcium signal indicator and used for imaging calcium dynamic signals. In addition, *Rosa26-LSL- tdTomato* mice were bred with *Piezo1* iKO to label hair follicles and used for hair cycle tracking by live animal imaging. For PIEZO1 activation in the skin, Yoda1 (100 μl of 100 μM) was ectopically applied on the ear skin, and calcium imaging was performed following the treatment.

### Immunostaining and imaging

For analysis of back skin phenotypes, 10-μm optimal cutting temperature (OCT) compound embedded sections were fixed in 4% paraformaldehyde for 10 min in phosphate-buffered saline (PBS) and washed three times in 1× PBS on shaker for 5 min at room temperature. Then, sections were blocked with 2.5% normal donkey serum and 2.5% normal goat serum in PBS. Sections were incubated with primary antibodies overnight at 4°C. After incubation with primary antibodies, sections were washed three times in 1× PBS for 5 min and incubated for 1 hour at room temperature with Alexa Fluor 594–, Alexa Fluor 555–, Alexa Fluor 488–, or Alexa Fluor 647–conjugated secondary antibodies (1:2,000; Invitrogen/Molecular Probes). Alexa Fluor 488–conjugated phalloidin (1:50; A12379; Invitrogen) or Alexa Fluor Plus 555 Phalloidin (1:200; A30106; Invitrogen) were used to stain F-actin. For pMLC2 staining, fluorescent signals of pMLC2 were amplified for 10 min and detected using the TSA Plus Fluorescein System (PerkinElmer) by following the manufacturer’s instruction. Nuclei were stained with Hoechst 33342 (1:5000; Invitrogen).

The following primary antibodies were used: anti-Ki67 antibody (1:500; Abcam, ab15580), NFATc1 (7A6) antibody (1:200; Santa Cruz Biotechnology, sc-7294), CD34 monoclonal antibody (RAM34) (1:200; eBioscience, 14-0341-82), c-Jun(60A8) rabbit mAb (1:200; Cell Signaling Technology, 9165T), KLF6 antibody (1:200; Novus Biologicals, NBP3-05013), recombinant anti-thrombospondin 1 antibody (1:200; Abcam, ab263905), phospho-myosin light chain 2 (Thr^18^/Ser^19^) (E2J8F) (1:50; Cell Signaling Technology, 95777S), and YAP (D8H1X) XP rabbit mAb (1:50; Cell Signaling Technology, 14074).

PLA was performed according to the published protocol (*66*) and the manufacturer’s instruction. The primary antibodies were used as follows:

E-cadherin and b-Catenin PLA staining: anti–E-cadherin antibody (1:500; BD Biosciences, 610181), anti–b-Catenin (Sigma-Aldrich, catalog no. C2206).

E-cadherin and Piezo1-TdTomato PLA staining: anti–E-cadherin antibody (1:500; BD Biosciences, 610181), and anti–red fluorescent protein antibody (Rockland, 600-401-379).

Imaging was performed on Nikon W1 Dual CAM Spinning Disk at Northwestern University Feinberg School of Medicine.

### scRNA-seq analysis

Single-cell sequencing analysis was performed using Seurat (v5.0.3) (*67*). Raw cell by barcode matrices corresponding to each dataset were loaded into Seurat and merged before quality control and filtering. High-quality cells—defined as containing between 1500 and 5000 unique genes, less than 30,000 unique molecular identifiers (UMIs), and less than a 10% mitochondrial read percentage—were retained for downstream analysis. Cell cycle scoring was performed from the log-normalized counts for each dataset using the Seurat CellCycleScoring() function, followed by SCTransform (v0.4.1) (*68*) normalization using 3000 variable genes. Integration of the control and Piezo1 iKO 2 datasets was performed using Seurat’s canonical correlation analysis implementation, followed by dimensionality reduction with PCA. Uniform Manifold Approximation and Projection (UMAP) embedding and nearest neighbor identification were performed using the first 40 principal components. Louvain clustering was performed using the Seurat FindMarkers() function using values ranging from 0.5 to 1.0 for the resolution parameter, with a resolution of 0.6 used for the final analysis. Cell types were annotated on the basis of the expression of canonical marker genes. Differentially expressed genes between control and each Piezo1 iKO treatment group were identified via using the Wilcoxon rank sum test implemented in the Seurat FindMarkers() function. Genes with an average log2fold change > ±0.25 and an adjusted *P* value < 0.05 were considered significantly differentially expressed. Gene Ontology term analysis was performed using the clusterProfiler (v4.6.2) R package (*69*).

### Calculating enrichment of NFATC1 target genes

We assessed the effect of our Piezo1 iKO on known NFATC1-modulated genes using our previously published bulk RNA-seq analysis of FACS-sorted (Sca1lo CD34hi ITGA6hi) *Nfatc1* cKO and control hair follicle stem cells (HF-SCs) (*35*). AUCell (v1.20.2) (*70*) was used to compute a per cell area under the recovery curve statistic for each set of significantly down- and up-regulated genes (adjusted *P* value < 0.05) from the raw count matrix for each treatment group. A Kruskall-Wallis test with a post hoc pairwise Wilcoxon rank sum test, implemented in R, was used to test for significant differences in AUCell scores between treatment groups.

### scATAC-seq data analysis

scATAC-seq analysis was performed using our previously published 1-year-old telogen scATAC-seq data. scATAC-seq analysis was performed using ArchR (v1.0.2) (*71*). Poor quality cells with <1000 fragments and a TSS ratio < 4 were removed before downstream analysis. Putative doublet cells were identified and removed using the ArchR addDoubletScores() and filterDoublets() functions, respectively. Dimensionality reduction was performed using ArchR’s iterative latent semantic indexing (LSI) approach using two rounds of iteration, with an initial clustering resolution of 0.2. A UMAP embedding was calculated for the scATAC-seq data using the ArchR addUMAP() function with the number of nearest neighbors set to 30. Louvain clustering with a resolution of 0.6 was used to identify cell clusters. Cluster identity was defined using both ArchR gene activity scores and through integration of the activity score matrix with our scRNA-seq control data. MAGIC (*72*)–imputed gene scores were used to visualize the accessibility of select marker genes. Peak calling for the HF-SCs cluster was performed using pyCisTopic (*73*) (v2.0a0). Following cell-type annotation, fragments were pseudobulked by cell type/cluster using the PyCisTopic export_pseudobulk() function, and peaks for each cluster were called using MACS2 (v2.2.9.1) (*74*) via the pyCisTopic peak_calling() function using the default parameters.

### Motif analysis of Piezo1 iKO DEGs in HF-SCs

HOMER (v4.11.1) (*75*) was used to identify enriched motifs within a 20-kB window surrounding the transcriptional start site of each significantly differentially expressed gene. First, an R pipeline was used to generate a bed file containing the genomic locations of the 10-kb regions upstream and downstream of the transcriptional start site for each identified DEG within the mouse mm10 genome. Bedtools (v2.27.1) (*76*) was then used to intersect each region with the consensus peaks identified in the scATAC-seq HF-SCs cluster to obtain the peaks falling within each TSS 20-kb window. Motif enrichment analysis was performed using the Homer findMotifsGenome.pl program with analysis restricted to known motifs. Following motif enrichment analysis, selected motifs were annotated using the Homer annotatePeaks.pl program and intersected with the DEG TSS windows to identify peaks falling within each gene.

### CUT&RUN

CUT&RUN was performed using the CUTANA ChIC/CUT&RUN Kit (EpiCypher no. 14-1048) and following the manufacturer’s protocol with minor modifications. In brief, mouse HF-SCs were collected and resuspended in 100 μl of wash buffer [20 mM Hepes (pH 7.5), 150 mM NaCl, and 0.5 mM spermidine supplemented with protease inhibitor EDTA-free tablet (Thermo Fisher Scientific, A32965)]. FACS purified HF-SCs (K14-H2bGFP^hi^/Sca1^neg^/CD34^hi^/CD49f^hi^) from P49 telogen hair follicles were then incubated with activated ConA beads for 10 min at room temperature. Bead-bound cells were resuspended in antibody targeting c-JUN (1:15; Cell Signaling Technology, no. 9165) overnight on a nutator at 4°C with the antibody buffer (wash buffer + 0.01% digitonin and 2 mM EDTA) supplemented with 0.1% bovine serum albumin, 100 nM trichostatin A, 0.1 U of citrate synthase, and 1 mM oxaloacetic acid. The following day, cells were washed with cell permeabilization buffer (wash buffer + 0.01% digitonin) twice and incubated with Proteins A and G to Micrococcal Nuclease (pAG-Mnase) for 1 hour on a nutator at 4°C. After incubation, cells were washed, 100 mM calcium chloride was added to each sample while on ice, and samples were incubated for 2 hours on a nutator at 4°C. Last, cells were incubated at 37°C for 30 min for DNA release, and DNA was purified using SPRI magnetic beads. CUT&RUN library was prepared from CUT&RUN DNA with NEBNext Ultra II DNA Library Prep Kit for Illumina (E7645), and paired-end DNA sequencing was performed on a NovaSeq platform.

### Intravital live imaging and image processing

Intravital live imaging was performed as previously described (*77*, *78*) with modifications as noted (*14*). Mice used for imaging were sedated using isoflurane (500 mg/liter) mixed with air. Once a mouse was fully sedated, it was put on a warm pad at 37°C. Isoflurane (200 mg/liter) mixed with air were maintained during imaging. Night-time ointment (Genteal, NDC 0078-0473-97) was applied to keep eyes moisturized. Custom-manufactured spatula was used to flatten the region of interest (ROI) and maintained at adjustable height. Double-sided tapes were used to adhere the lower side of ear onto the spatula. After applying long-lasting Genteal gel (0078-0429-47) to the region of interest, a second adjustable spatula, glued with glass cover on one end, was gently pressed down to the ear so that the cover glass was right on top of the region. Long-lasting Genteal was applied on the glass cover to keep the tip of objective merged in during imaging. For resonant and non-descanned detection (NDD) imaging, the Leica DiveB Sp8 Multiphoton imaging system was used with a MaiTai laser tuning to 920 nm to capture both GCaMP6f and tdTomato signals. Scanning resolution was set to 1024 × 1024. Zoom factor was set to 1.25 for larger regions and 3.00 for smaller regions. For resonant imaging, a single *z* plane was scanned every 2 s for a total of 5 min for epidermal movies and for a total of 10 min for HF movies. To increase signal intensity, line accumulation was set to 8. For NDD imaging, the time lapse images of GCAMP6f-tdTomoto were scanned from the epidermis to HFs with a *z* depth of 80 to 120 mm once every 2 min. After the image session is done, the mouse was kept in air to recover before sending back in cage. Two photon images were acquired using LAS X software from Leica. The time-lapse images were aligned using Fiji > plugins > registration > descriptor-based series registration (2d/3d +t) before exported. The exported tif files were further converted to Imaris file format using Imaris File Converter software. To track the hair cycle progression, an Olympus FVMPE-RS multiphoton imaging system was applied for acquiring K5-CreER/TdTomato (control and Piezo1 iKO) hair follicle images. An Insight X3 laser was tuned to 1050 nm for tdTomato excitation. Objectives (10×) were used for images. Two-photon images were acquired using FluoView software from Olympus. The Imaris ×64 9.7.2 were used for further analysis. Movies were generated from Imaris.

### E-cadherin microbead experiment and force calibration

Primary keratinocytes were maintained in a low Ca^2+^ medium. For the micropipette aspiration experiments, primary keratinocytes were seeded in a homemade chamber coated with fibronectin (10 μg/ml). To enable membrane expression of E-cadherin, a final concentration of 2 μM Ca^2+^ was applied. After overnight incubation, E-cadherin–coated microbeads (6.25 ± 0.87 μm in diameter) were added to the chamber and preincubated with the cells for 3 hours, enabling the preformation of E-cadherin adhesion between beads and cells. To minimize the formation of many E-cadherin dimers, we allowed the beads to sink to the cell surface under a small gravitational force (~0.04 pN) rather than actively pressing the bead against the cell surface. The calcium indicator dye Fluo-4 Direct (F10471, Thermo Fisher Scientific) with the dilution of 1:8 according to the manufacturing instruction was then applied and incubated at 37°C for 1 hour before experiments for the detection of calcium flux. During the experiments, a glass micropipette controlled with a mortised controller (*29*) was used to apply force to E-cadherin via E-cadherin–coated microbeads and to probe E-cadherin–dependent Piezo1 activation. To verify that the beads formed E-cadherin adhesions with the cells, a minor positive pressure was applied to gently blow away nonadhered beads (see Fig. 3D).

Forces in the piconewton range were achieved by applying a negative pressure to a hollow micropipette positioned about 5 μm from the microbead (see Fig. 3D), which exerted forces of approximately 20 pN (see Fig. 3F). The force was calibrated based on the speed (*v*) of the microbead moving toward the micropipette tip using the Stokes equation F=6πηrv, where $\eta =10^{-3}$ Pa·s and *r* = 3 μm are the viscosity of the medium and the radius of the microbead, respectively. To achieve a force of ~20 pN, the speed of the microbead was controlled to approximately 300 μm/s, maintained by adjusting the negative pressure applied to the microbead.

The calcium influx was monitored and measured during the bead capture process. To study the calcium influx response by PIEZO1 activation, Yoda1 at a concentration of 30 μM, was added to the chamber. To study the specificity of PIEZO1 activation–dependent calcium influx by aspiration of E-cadherin beads, the PIEZO1 inhibitor GsMTx4, at a concentration of 1 μM, was added to the chamber. Non–E-cadherin–coated beads were used to ensure the specificity of E-cadherin adhesion that triggers the PIEZO1-dependent calcium influx. Calcium influx signal was also recorded by micropipette scratching on the cells. The ratio of calcium increase was measured using the equation (*I*_c_ − *I*_0_)/*I*_0_, where *I*_0_ represents the calcium intensity before bead capture and *I*_c_ denotes the recorded real-time calcium signal after bead capture.

### Atomic force microscopy

Tissue stiffness was measured according to previously described protocols (*14*). Briefly, OCT cryosections of back skin, 25-μm thick, from both control and Piezo1 iKO samples were prepared to measure the stiffness of hair follicles and dermal tissue. Samples were affixed to Superfrost Plus Adhesion Microscope slides and washed three times for 5 min in 1× PBS at room temperature to remove OCT.

For AFM measurements, the AFM-based nanoindentation measurements were carried out using a commercial AFM (JPK Nanowizard) equipped with a Nikon optical microscope. AFM nanoindentation tests were performed using a 5-μm-radius cylindrical tipped nitride cantilever (SAA-SPH-5UM, Bruker) in 1× PBS. Cantilever spring constants were calibrated each time before sample measurements using the thermal fluctuation method, which were in the range of 0.20 to 0.30 N m^−1^. During measurements, samples were maintained in 1× PBS. Brightfield and Rosa-TdTomato images were captured and used to align the cantilever to the sample and for image co-registration. Two-dimensional (2D) force maps were taken in 15 μm by 15 μm grids with 64 sample points per axial dimension. AFM measurements were made using a cantilever deflection set point of 2.0 nN and an indentation rate of 5 μm s^−1^ to capture elastic properties and minimize viscoelastic effects.

The force-indentation traces were analyzed to obtain the Young’s modulus of the cells by using the JPK data processing program. After baseline correction and contact point estimation, the approaching force-indentation curve was fitted with the Hertz (Spherical) model (Eq. 1). Constant parameters were chosen to minimize the bias for different samples (*79*)

$$F\left(x\right)=\frac{4}{3}\frac{E}{\left(1-\upsilon^{2}\right)}\sqrt{r}x^{3/2}$$ (1)

where F is the force of the cantilever, x is the indentation distance of the cell pressed by the cantilever, E is the Young’s modulus of the cell layer, r is the radius of the spherical indenter, and υ is the Poisson ratio. The Poisson ratio of cell is normally in the range of 0.3 to 0.5. We chose υ=0.5 in all calculations.

### Quantification and statistical analysis

#### Quantification of GCaMP6f:tdTomato signals for resonant imaging

Step 1: If necessary, drift correction of live-imaging movies was performed via ImageJ.

Step 2: After drift correction, cells were manually segmented. For epidermal cells, only cells that experienced high-intensity fluorescence events during imaging were segmented. For HF cells, all cells that were sufficiently in focus were segmented. Manual segmentation of cells into ROIs was performed with the ImageJ freehand selection tool. For each HF movie, 8 to 10 ROIs from the empty regions surrounding hair follicles were separately measured to represent levels of background signal.

Step 3: For epidermal cells, GCaMP6:tdTomato ratios were calculated according to Eq. 2

$$r=\frac{c_{\left(\text{GCaMP}6f,t\right)}}{c_{\left(\text{tdTomato},t\right)}}$$ (2)

where *c* is the mean gray value of a given cell. For hair follicle cells, normalized GCaMP6f:tdTomato ratios (*r*) were calculated according to Eq. 3

$$r=\frac{c_{\left(\text{GCaMP}6f,t\right)}-\bar{b_{\left(\text{GCaMP}6f,t\right)}}}{c_{\left(\text{tdTomato},t\right)}-\bar{b_{\left(\text{tdTomato},t\right)}}}$$ (3)

where $\bar{b}$ is the average of the mean gray values of the 8 to 10 background ROIs, *t* is time, and *c* is the mean gray value of a given cell.

Step 4: For a given cell, the baseline GCaMP6f:tdTomato ratio, $r_{0}$, was calculated as the average of values of *r* in the lower 50th percentile for that cell. To calculate the instantaneous fold change for a given cell at time *t* above the baseline, we calculated $\frac{r_{t}}{r_{0}}$*.*

#### Quantification of GCaMP6f:tdTomato signals for NDD imaging

Step 1: Drift correction of live-imaging movies was performed via ImageJ.

Step 2: After drift correction, individual hair follicles with sufficiently high resolution of cells and low drift were selected for quantification. Each individual hair follicle was segmented at a representative *z* value with the greatest number of cells in focus. Manual segmentation of cells into ROIs was performed with the ImageJ freehand selection tool. During segmentation, care was taken to ensure that no ROIs overlapped the region of the hair shaft, which is highly autofluorescent. We discarded any frames of movies where the hair follicle appeared significantly misaligned with segmented ROIs due to sudden drift. For each movie, six to eight ROIs from the empty regions surrounding hair follicles were separately measured to represent levels of background signal.

Step 3: Normalized GCaMP6f:tdTomato ratios were calculated according to Eq. 4

$$\frac{r_{\left(\text{GcCamp}6,t\right)}-\bar{b_{\left(\text{GcCamp}6,t\right)}}}{r_{\left(\text{TdTomato},t\right)}-\bar{b_{\left(\text{TdTomato},t\right)}}}$$ (4)

where $\bar{b}$ is the average of the mean gray values of the six to eight background ROIs, *t* is time, and *r* is the mean gray value of a given ROI.

Step 4: Because of upward drift of the ear during imaging or photobleaching, GCaMP6f:tdTomato ratios often demonstrated an upward linear trend with respect to time. To correct for this effect, we calculated linear regression models for each individual ROI with respect to time such that each data point can be represented with Eq. 5

$$y_{i}=\alpha +\beta i+\varepsilon_{i}$$ (5)

where $y_{i}$ is the normalized GCaMP6f:tdTomato ratio for a given ROI at time point i, α is the *y* intercept of the linear regression line, β is the slope of the linear regression line, and $\varepsilon_{i}$ is an error term. From this linear regression, we calculated the drift-corrected GCaMP6f:tdTomato ratio f at time t as Eq. 6

$$f_{t}=\alpha +\beta \left(1\right)+\varepsilon_{t}$$ (6)

Step 5: For a given time point and ROI, Δfis the difference between *f* and the ROI’s baseline, defined to be the average of the lower 10th percentile of *f* across all time points for that ROI. Hence, Δ𝑓 represents the “height” of signal intensity above a ROI’s baseline. Cumulative signal intensity is the sum of all Δ𝑓 values across all time points for a given ROI. As not all the movies have an equal number of time points, we calculated estimated cumulative signal intensity by first dividing the cumulative signal intensity by the number of time points and then multiplying the resulting number by 30 to produce an estimate of cumulative influx observed during a 30-min movie. For comparing high-intensity flashing events in the epidermis and hair follicle, we calculated maximum fold change, defined for a given ROI as its maximum value of *f* divided by the aforementioned baseline. All data analysis was performed in R.

#### Quantification and 3D F-actin versus YAP analysis

To quantify the immune-staining signals of YAP and cortical F-actin in HGs, the images were converted to Imaris for further quantification. Briefly, Imaris Surface module was applied to automatically select YAP positive areas in the nucleus in HG cells. The mean/median intensity of YAP signals of the selected region was obtained from the statistical tab. Cortical F-actin signals were quantified using line quantification, crossing F-actin bundles, in Fiji software. The mean peak intensities of five line quantifications per cell was calculated.

After quantification of F-actin and YAP signals in wild-type, control, and Piezo1iKO samples, we did normalization of F-actin values and YAP values by dividing all the values with the maximum value of the condition. For wild-type data, we also assigned a phase of the hair cycle (telogen and anagen) to each cell depending on their morphology and hair cycle stages. We then defined telogen center, *t*, and anagen center, *a*, by calculating the mean of normalized F-actin value and YAP value for telogen and anagen cells in wild type, respectively. We then defined a binary phase score for a cell *c* as

Binary phase score=dist(c,a)−dist(c,t)

where the distance is between normalized F-actin and YAP of the cell and two centers.

Observe that if a cell is closer to the telogen center, then it will have a positive binary phase score. Otherwise, if it is closer to the anagen center, then it will have a negative binary phase score. If a cell is equidistant from both centers, then it will have a zero score.

For visualization, we defined a telogen-anagen phase landscape by finding binary phase scores for simulated normalized F-actin and YAP value combinations (*n* = 21^2^, where both values in any combination is out of a sequence ranging from 0 to 1, incremented by 0.05) and created a smoothed 3D surface from simulated combinations with

x=normalizedYAPvalue

y=normalizedF−actin value

z=binary phase score

Later, for each cell in control and Piezo1iKO samples, we again calculated its binary phase score based on the normalized YAP and F-actin values and then plotted the data to the telogen-anagen phase landscape. For each group of data, one-sided Wilcoxon test was used to test the difference in mean.

To investigate the significance of the correlation between YAP and cortical F-actin [Phalloidin (PHALL) signal], we conducted a randomization test by shuffling all YAP and PHALL value pairs 1000 times, and we calculated *R*^2^ (coefficient of determination) with 1000 for each shuffle and built a background distribution from these 1000 *R*^2^. Last, we defined the empirical *P* value of *R*^2^ as $\begin{array}{c} P\;{\text{value}}_{\text{emperical}}=\frac{\#\text{of shuffles with}\;R^{2}>R_{\text{YAP}-\text{PHALL}}^{2}+1}{1000+1} \end{array}$, where 1 is a pseudocount.

For other cortical F-actin signal quantification, more than 10 line quantifications were used for the one HG area, and they were aligned by the peak values of those line quantification. The mean intensity of F-actin signals for each pixel was used for quantification.

#### Statistical analysis

The scRNA-seq was performed with control and Piezo1 inducible KO samples at the same time on the same chip to reduce the batch effect. All experiments were designed such that there were always littermate controls. All statistical tests performed are as indicated in the figure legends. No statistical methods were used to predetermine sample size. The experiments were not randomized, and the investigators were not blinded to allocation during experiments and outcome assessment, except where stated.

For all experiments with error bars, the SD was calculated to indicate the variation within each experiment. Numbers of animals used for phenotype study has indicated in the manuscript and figure legends. Student’s *t* test or Mann-Whitney *U* test was used for most experiments, as indicated in the figure legend.
