# Supplementary material for: PIEZO1-mediated calcium signaling reinforces mechanical properties of hair follicle stem cells to promote quiescence
Source: Sci Adv. 2025 May 28;11(22):eadt2771. doi: 10.1126/sciadv.adt2771 (PMC12118625; doi:10.1126/sciadv.adt2771)
Supplement: Supplementary file 1 — Figs. S1 to S10 Legends for data S1 to S6 Legends for movies S1 to S10 [file sciadv.adt2771_sm.pdf]

Science Advances
AAAS

Supplementary Materials for

PIEZO1-mediated calcium signaling reinforces mechanical properties of hair follicle stem cells to promote quiescence

Jingjing Wang *et al.*

Corresponding author: Rui Yi, yir@northwestern.edu

Sci. Adv. **11**, eadt2771 (2025)
DOI: 10.1126/sciadv.adt2771

The PDF file includes:

Figs. S1 to S10
Legends for data S1 to S6
Legends for movies S1 to S10

Other Supplementary Material for this manuscript includes the following:

Data S1 to S6
Movies S1 to S10

Figure S1

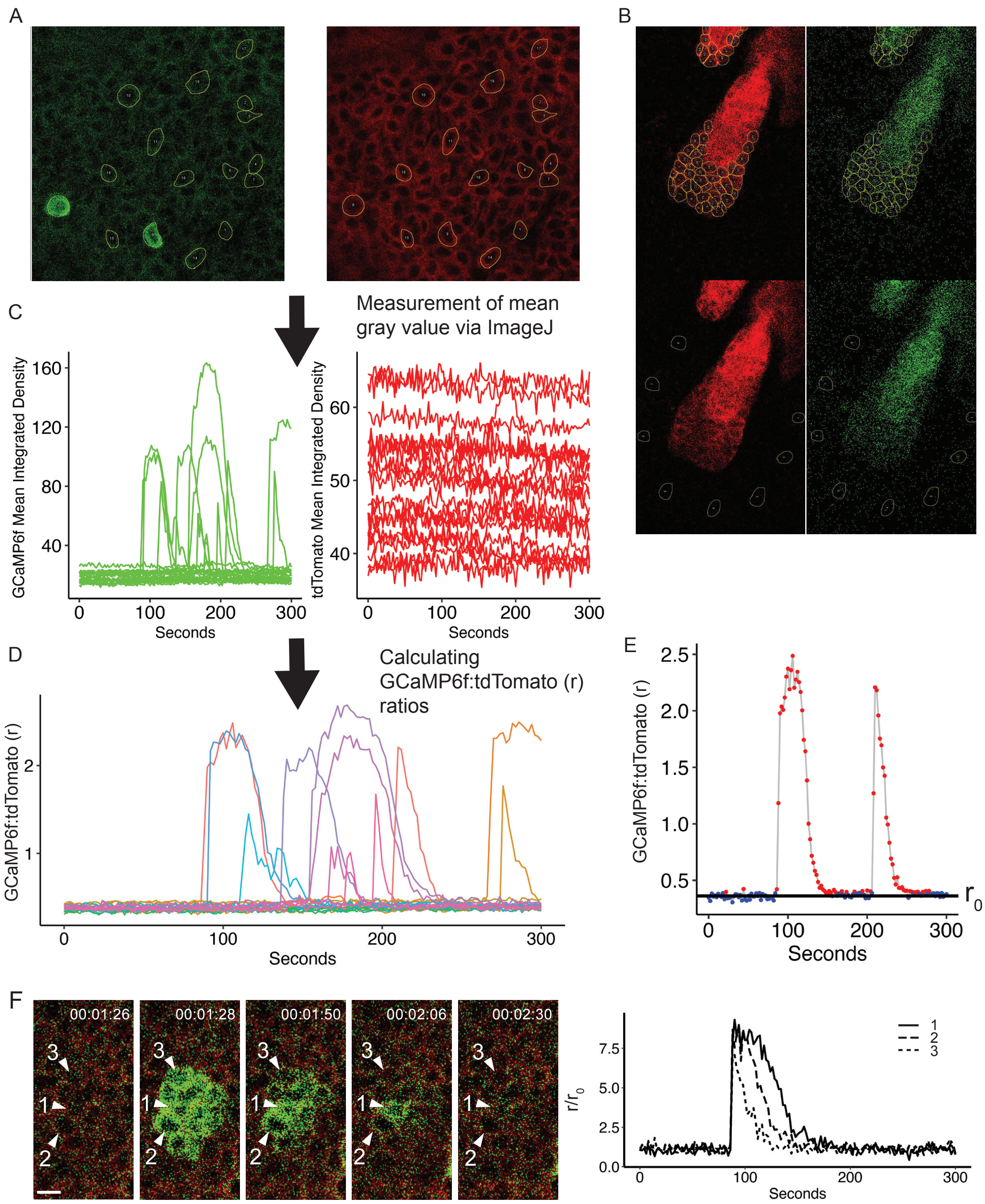
A
B
C
Measurement of mean gray value via ImageJ
GCaMP6f Mean Integrated Density
160
120
80
40
0
100
200
300
Seconds
tdTomato Mean Integrated Density
60
50
40
0
100
200
300
Seconds
D
Calculating GCaMP6f:tdTomato (r) ratios
GCaMP6f:tdTomato (r)
2
1
0
100
200
300
Seconds
E
GCaMP6f:tdTomato (r)
2.5
2.0
1.5
1.0
0.5
0
100
200
300
Seconds
r0
F
00:01:26
00:01:28
00:01:50
00:02:06
00:02:30
1
2
3
r/r0
7.5
5.0
2.5
0.0
0
100
200
300
Seconds

Fig. S1: Workflow of ratiometric calcium quantification for resonant imaging

A, Manual segmentation of selected epidermal cells in both GCaMP6f (left) and tdTomato (red) channels in ImageJ. **B**, Manual segmentation of bulge HF-SCs and HG cells of each hair follicle (top) and background regions around the hair follicle (bottom) in ImageJ. Background measurements are used to estimate noise and subtracted from GCaMP6f and tdTomato channel measurements. **C**, Mean values of each cell segmented in (**A**) in both GCaMP6f (left) and tdTomato (right) channels with a 2-second interval over the course of 5 minutes. **D**, Calculation of GCaMP6f:tdTomato ratios, *r,* over 5 minutes. **E**, The baseline level of *r* for each cell, r_0, is calculated as the average of all data points in the lower 50th percentile of *r* (blue) for that cell. **F**, A representative, high-intensity calcium spike coordinated across several cells during five time points in the epidermis. A plot of GCaMP6f:tdTomato fold change as a function of time for three cells, numbered as 1, 2 and 3, reveals one distinct, coordinated calcium spike event over the course of 5 minutes.

Figure S2

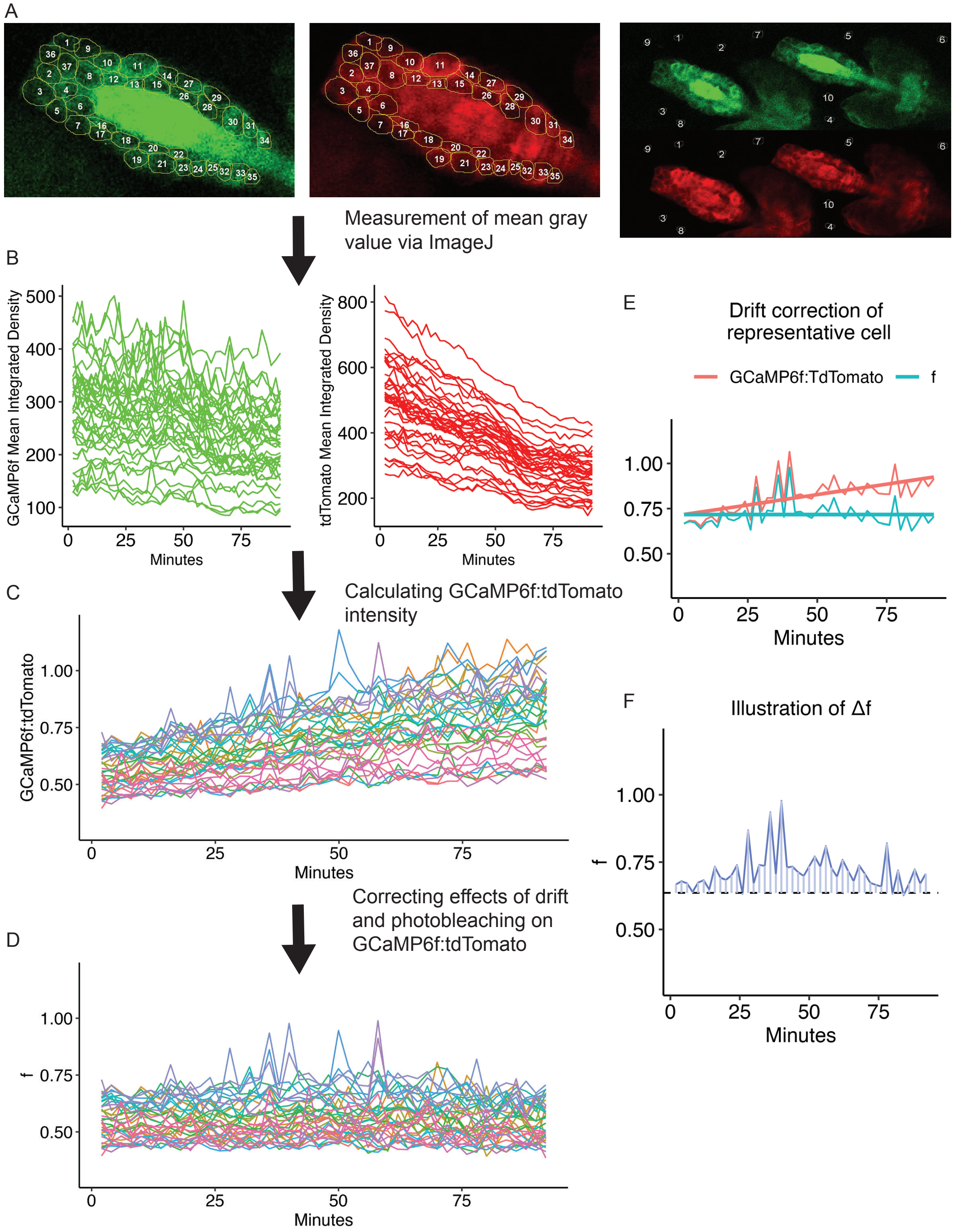
A
Measurement of mean gray value via ImageJ
B
GCaMP6f Mean Integrated Density
tdTomato Mean Integrated Density
Minutes
C
Calculating GCaMP6f:tdTomato intensity
GCaMP6f:tdTomato
Minutes
D
Correcting effects of drift and photobleaching on GCaMP6f:tdTomato
f
Minutes
E
Drift correction of representative cell
GCaMP6f:TdTomato
f
Minutes
F
Illustration of Δf
f
Minutes

Fig. S2: Workflow of ratiometric calcium quantification for NDD imaging

A, Segmentation and quantification of HF-SC and HG cells. ImageJ is used to manually segment bulge HF-SC and HG cells of each hair follicle (left) and background regions around the hair follicle (right). **B**, Mean values of each cell for both GCaMP6f (left) and tdTomato (right) channels are acquired at a 2-min interval for 90 minutes. During imaging sessions where sample drift or photobleaching occurred, mean values from both channels demonstrated approximately linear downward trends. **C**, Calculation of GCaMP6f:tdTomato ratios over the course of 90 minutes. **D**, Correction of drift and photobleaching effects on GCaMP6f:tdTomato ratios through linear regression to obtain *f*. **E**, Correction of drift and photobleaching effects on GCaMP6f:tdTomato ratios through linear regression for a representative cell. **F**, Illustration of parameters of quantification. *Δf* at a given time point and cell is defined as the difference between *f* and the average of the lower 10th percentile of *f* across all time points for that cell. Cumulative calcium spike for a given cell is defined as the sum of all *Δf* values across all time points.

Figure S3

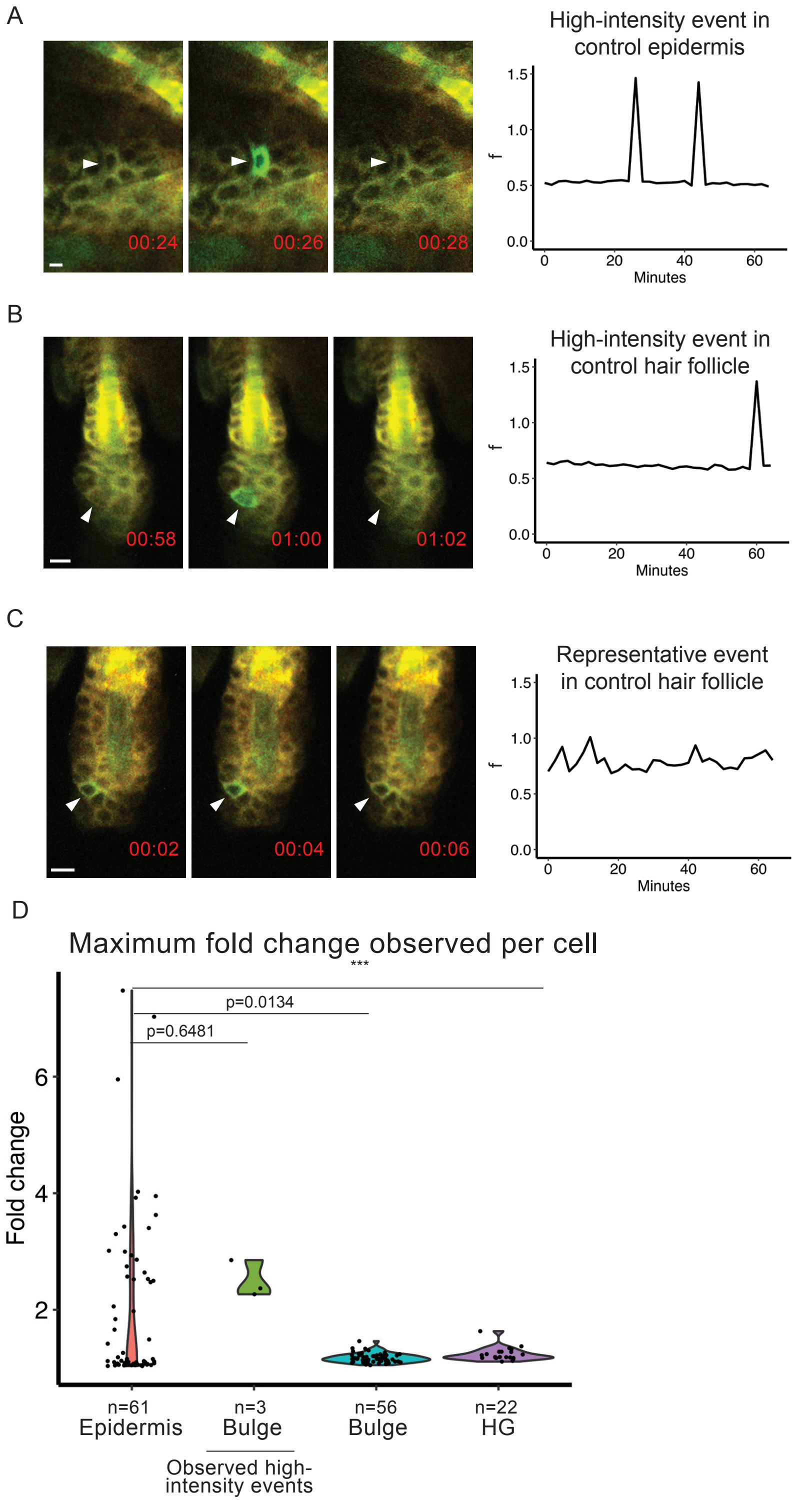
A
00:24
00:26
00:28
High-intensity event in control epidermis
f
Minutes
B
00:58
01:00
01:02
High-intensity event in control hair follicle
f
Minutes
C
00:02
00:04
00:06
Representative event in control hair follicle
f
Minutes
D
Maximum fold change observed per cell
Fold change
***
p=0.0134
p=0.6481
n=61
Epidermis
n=3
Bulge
n=56
Bulge
n=22
HG
Observed high-intensity events

Fig. S3: Frequent and weak calcium spikes in the hair follicle are distinct from rare, epidermal-like high-intensity events.

A, A representative, high-intensity calcium spike across three consecutive time points in the epidermis. A plot of *f* (drift-adjusted GCaMP6f:tdTomato) as a function of time illustrates two distinct flashing events over the course of 60 minutes within the highlighted cell. Scale bar, 10 µm. **B**, A representative, high-intensity calcium spike across three consecutive time points in a cell in the bulge region of a hair follicle. A plot of *f* as a function of time illustrates one distinct flashing event over the course of 60 minutes within the highlighted cell. Scale bar, 10 µm. **C**, Weaker calcium spikes in a cell in the bulge region of a hair follicle across three consecutive time points. A plot of *f* as a function of time illustrates frequent, mild flashing events over the course of 60 minutes within the highlighted cell. Scale bar, 10 µm. **D**, The maximum fold changes recorded during the rare high-intensity events observed in hair follicles fall within the range of maximum fold changes observed in epidermal cells. In comparison, the majority of cells in the bulge and HG region show much smaller maximum fold changes. P values were determined by Mann-Whitney U test.

Figure S4

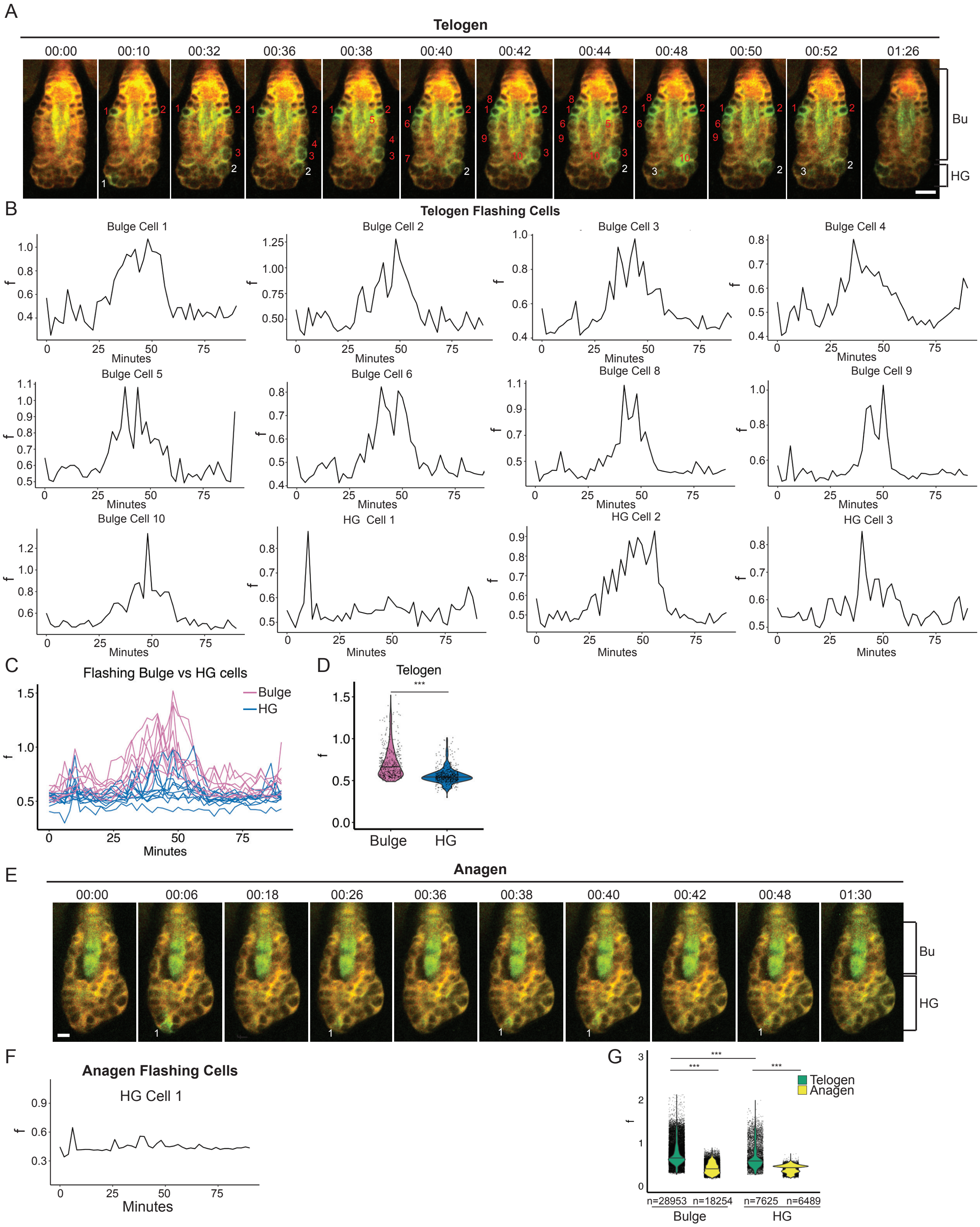

A
Telogen
00:00
00:10
00:32
00:36
00:38
00:40
00:42
00:44
00:48
00:50
00:52
01:26
Bu
HG
B
Telogen Flashing Cells
Bulge Cell 1
Bulge Cell 2
Bulge Cell 3
Bulge Cell 4
Bulge Cell 5
Bulge Cell 6
Bulge Cell 8
Bulge Cell 9
Bulge Cell 10
HG Cell 1
HG Cell 2
HG Cell 3
Minutes
f
C
Flashing Bulge vs HG cells
Bulge
HG
Minutes
D
Telogen
***
Bulge
HG
E
Anagen
00:00
00:06
00:18
00:26
00:36
00:38
00:40
00:42
00:48
01:30
Bu
HG
F
Anagen Flashing Cells
HG Cell 1
Minutes
G
Telogen
Anagen
n=28953
n=18254
n=7625
n=6489
Bulge
HG

Fig. S4. HF-SC compartments display differential calcium dynamics during quiescence and activation

A, Representative images of calcium spikes (numbered) observed in the bulge (Bu) and hair germ (HG) compartments in telogen. Time lapse images, acquired at a 2-minute internal for 90 minutes, show the progression of the calcium spikes. Red labeled numbers indicate bulge cells with calcium spike events, white labeled numbers indicate HG cells with calcium spike events. Each image is annotated with the elapsed time from the start of the experiment, formatted as HH:MM (Hours:Minutes). Scale bar, 10 μm. **B**, Corrected calcium signals of labeled flashing bulge HF-SC and HG cells in (**A**) over 90 minutes. **C**, Calcium signal strength (*f*) of the representative bulge cells that demonstrate calcium spike events and all HG cells in telogen as shown in (**A**). Purple lines represent calcium signal strength for individual bulge cells. Blue lines represent calcium signal strength for individual HG cells. **D**, Calcium signal strength (*f*) of all events observed in HF-SC and HG cells in telogen as shown in (**A**). Statistical significance is determined by Mann-Whitney U Test (*** $P<0.001$). **E**, Representative images of calcium spikes (numbered) observed in the bulge (Bu) and hair germ (HG) compartments in early anagen. Time lapse images, acquired at a 2-minute internal for 90 minutes, show the progression of the calcium spikes. White labeled number (1) indicates a HG cell with calcium spike event. Scale bar, 10 μm. **F**, Calcium signal strength (*f*) of the labeled HG cell in anagen hair follicle (**E**). **G**, Calcium signal strength of all HF-SC and HG cells quantified in telogen and anagen. Total signal events are indicated by n numbers in the panel. Statistical significance is determined by Mann-Whitney U Test (*** $P<0.001$).

Figure S5

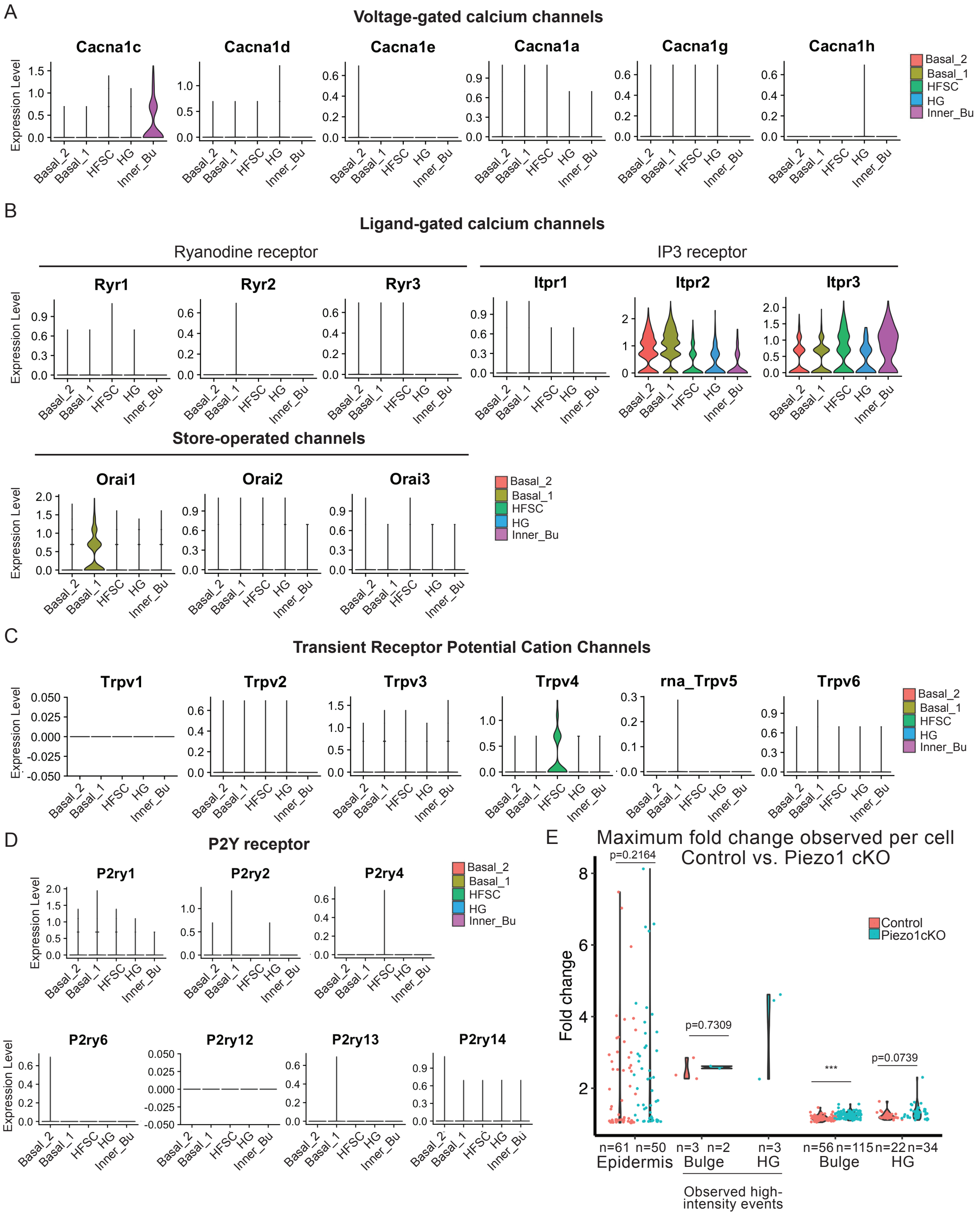

A
Voltage-gated calcium channels
Cacna1c
Cacna1d
Cacna1e
Cacna1a
Cacna1g
Cacna1h
Expression Level
Basal_2
Basal_1
HFSC
HG
Inner_Bu
B
Ligand-gated calcium channels
Ryanodine receptor
IP3 receptor
Ryr1
Ryr2
Ryr3
Itpr1
Itpr2
Itpr3
Store-operated channels
Orai1
Orai2
Orai3
C
Transient Receptor Potential Cation Channels
Trpv1
Trpv2
Trpv3
Trpv4
rna_Trpv5
Trpv6
D
P2Y receptor
P2ry1
P2ry2
P2ry4
P2ry6
P2ry12
P2ry13
P2ry14
E
Maximum fold change observed per cell
Control vs. Piezo1 cKO
Fold change
Control
Piezo1cKO
p=0.2164
p=0.7309
***
p=0.0739
n=61 n=50
Epidermis
n=3 n=2
Bulge
n=3
HG
n=56 n=115
Bulge
n=22 n=34
HG
Observed high-intensity events

Fig. S5. Expression patterns of calcium channels in epithelial cell populations

A, Expression patterns of voltage-gated calcium channels in major epithelial cell populations, including basal_1, basal_2, HFSC (bulge HF-SCs), HG (hair germ), Inner_Bu (Inner bulge niche cells). **B**, Expression patterns of ligand-gated calcium channels in major epithelial cell populations. **C**, Expression patterns of transient receptor potential cation channels in major epithelial cell populations. **D**, Expression patterns of P2Y receptor family members in major epithelial cell populations. **E**, Maximum signal fold change (f/f0) observed in epidermal cells and hair follicle cells. Events in hair follicle cells are classified as either rare, high-intensity events, or weaker, more frequent events. P values are determined by Mann-Whitney U test.

Figure S6

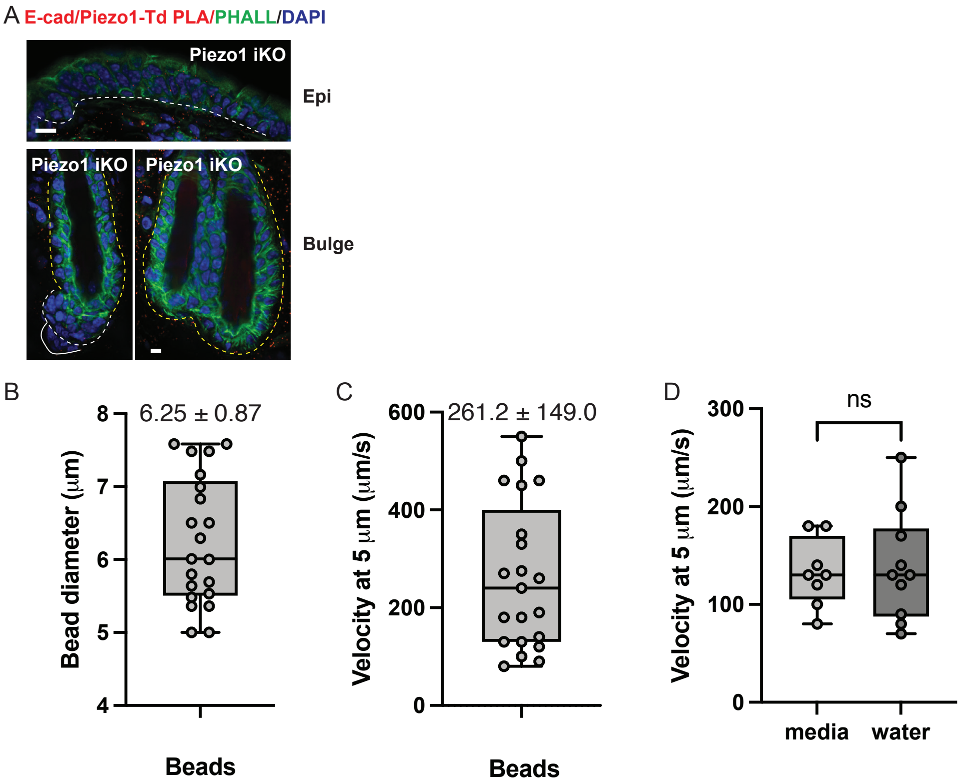

A E-cad/Piezo1-Td PLA/PHALL/DAPI
Piezo1 iKO
Epi
Piezo1 iKO
Piezo1 iKO
Bulge
B
6.25 ± 0.87
Bead diameter (μm)
8
7
6
5
4
Beads
C
261.2 ± 149.0
Velocity at 5 μm (μm/s)
600
400
200
0
Beads
D
ns
Velocity at 5 μm (μm/s)
300
200
100
0
media
water

Fig. S6. Supporting data for Figure 3

A, No PLA signals of Piezo1-tdTomato and E-cadherin are detected in *Piezo1* induced KO (iKO), serving as negative control for the PLA. Scale bar, 5 µm. **B,** The diameter of microbeads is ~6.25 µm. **C,** The average velocity and standard deviation of the microbeads at a distance of 5µm from the micropipette tips. **D,** The velocity of the microbeads at a distance of 5µm from the micropipette tip is the same between the culture medium and water, demonstrating the same viscosity of the medium and water.

Figure S7

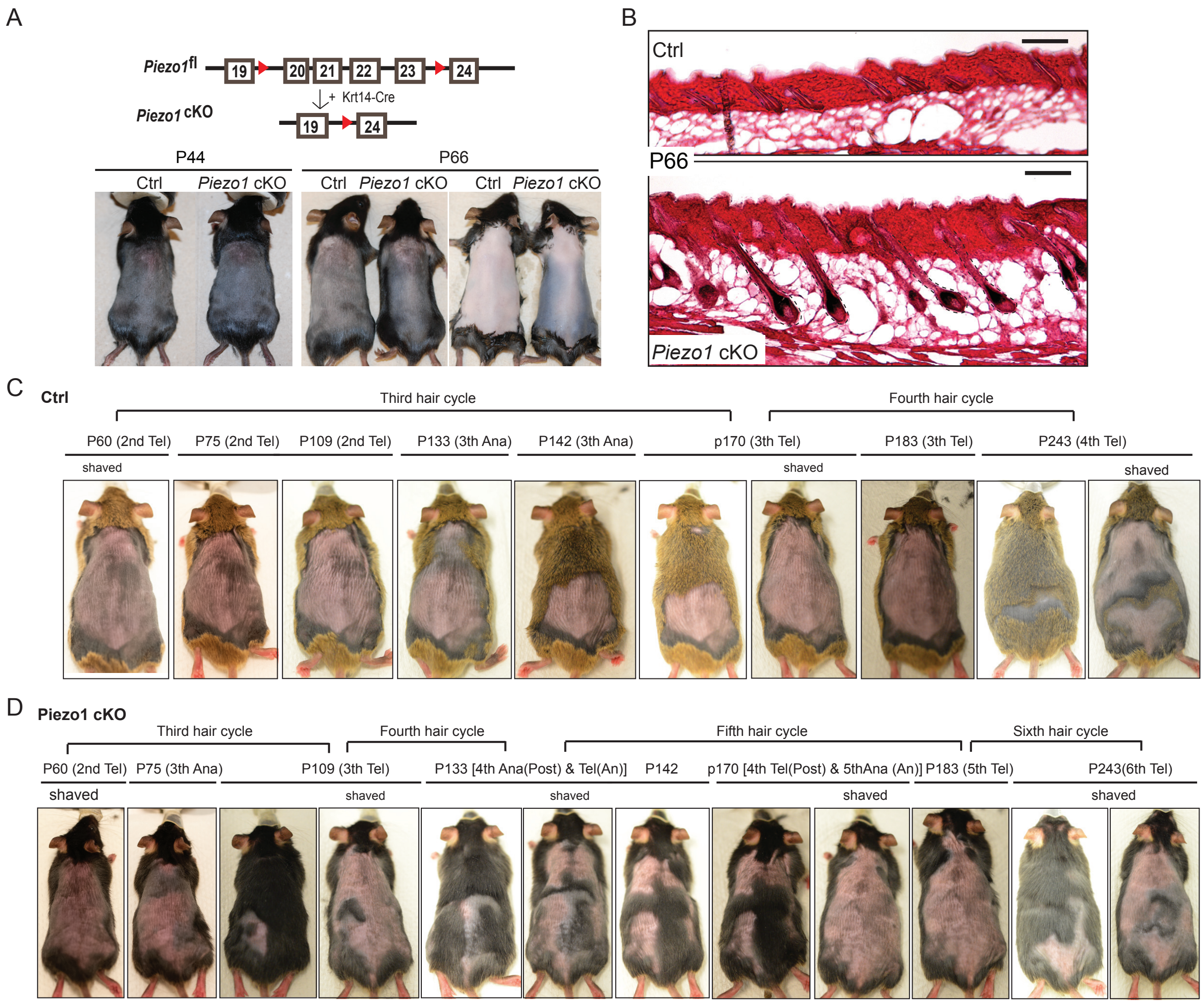

A
Piezo1fl
19
20
21
22
23
24
+ Krt14-Cre
Piezo1 cKO
19
24
P44
P66
Ctrl
Piezo1 cKO
Ctrl
Piezo1 cKO
Ctrl
Piezo1 cKO
B
Ctrl
P66
Piezo1 cKO
C
Ctrl
Third hair cycle
Fourth hair cycle
P60 (2nd Tel)
P75 (2nd Tel)
P109 (2nd Tel)
P133 (3th Ana)
P142 (3th Ana)
p170 (3th Tel)
P183 (3th Tel)
P243 (4th Tel)
shaved
shaved
shaved
D
Piezo1 cKO
Third hair cycle
Fourth hair cycle
Fifth hair cycle
Sixth hair cycle
P60 (2nd Tel)
P75 (3th Ana)
P109 (3th Tel)
P133 [4th Ana(Post) & Tel(An)]
P142
p170 [4th Tel(Post) & 5thAna (An)]
P183 (5th Tel)
P243(6th Tel)
shaved
shaved
shaved
shaved
shaved

Fig. S7. Deletion of *Piezo1* shortens the telogen length and promotes hair regeneration

A, Deletion of *Piezo1* in epithelial cells of the skin using *Krt14-Cre* promotes hair regeneration in the second hair cycle by P66. 6 pairs of animals are used for phenotypical analysis. **B**, H&E staining shows accelerated hair regeneration in *Piezo1* cKO. Scale bar, 100 μm. **C**, Phenotypical tracking of hair growth in control mice shows that most of control hair follicle in dorsal skin go through 4 hair cycles over 243 days. **D**, Phenotypical tracking of hair growth in *Piezo1* ckO mice shows that most of *Piezo1* cKO hair follicle in dorsal skin go through 6 hair cycles over 243 days.

Figure S8

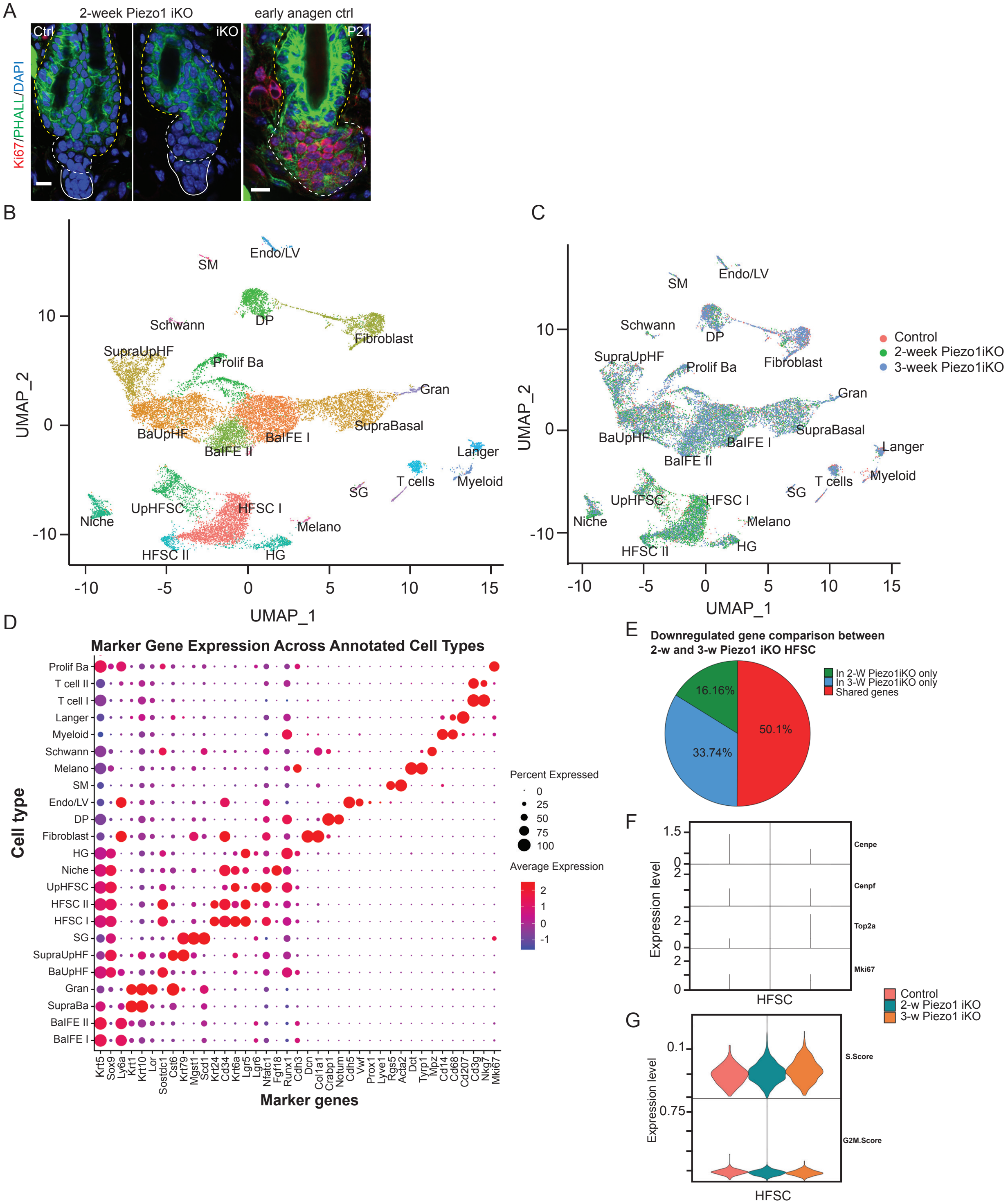

A
2-week Piezo1 iKO
early anagen ctrl
Ctrl
iKO
P21
Ki67/PHALL/DAPI
B
C
UMAP_1
UMAP_2
SM
Endo/LV
Schwann
DP
Fibroblast
SupraUpHF
Prolif Ba
Gran
BaUpHF
BalFE II
BalFE I
SupraBasal
Langer
T cells
Myeloid
SG
Niche
UpHFSC
HFSC I
HFSC II
HG
Melano
Control
2-week Piezo1iKO
3-week Piezo1iKO
D
Marker Gene Expression Across Annotated Cell Types
Cell type
Marker genes
Percent Expressed
Average Expression
Krt5 Sox9 Ly6a Krt1 Krt10 Lor Sostdc1 Cst6 Krt79 Mgst1 Scd1 Krt24 Cd34 Krt6a Lgr5 Lgr6 Nfatc1 Fgf18 Runx1 Cdh3 Dcn Col1a1 Crabp1 Notum Cdh5 Vwf Prox1 Lyve1 Rgs5 Acta2 Dct Tyrp1 Mpz Cd14 Cd68 Cd207 Cd3g Nkg7 Mki67
Prolif Ba
T cell II
T cell I
Langer
Myeloid
Schwann
Melano
SM
Endo/LV
DP
Fibroblast
HG
Niche
UpHFSC
HFSC II
HFSC I
SG
SupraUpHF
BaUpHF
Gran
SupraBa
BalFE II
BalFE I
E
Downregulated gene comparison between 2-w and 3-w Piezo1 iKO HFSC
In 2-W Piezo1iKO only
In 3-W Piezo1iKO only
Shared genes
16.16%
33.74%
50.1%
F
Expression level
Cenpe
Cenpf
Top2a
Mki67
HFSC
Control
2-w Piezo1 iKO
3-w Piezo1 iKO
G
Expression level
S.Score
G2M.Score
HFSC

Fig. S8. Single cell analysis of *Piezo1* iKO skin populations

A, Ki67 immunostaining shows that *Piezo1* iKO hair follicles are still in telogen after 2-week induction. An early anagen (P21) sample serves as the positive control, in which Ki67+ cells are detected in growing hair germ. Scale bar, 10 µm. **B**, UMAP plot of all cell populations of control and *Piezo1* iKO dorsal skin as detected by scRNAseq. **C**, Clustering of all cell populations remains similar after 2-week and 3-week induction of *Piezo1* deletion judging by scRNAseq datasets. **D**, Marker genes for major cell clusters detected in scRNAseq datasets. **E**, Pie chart for the comparison of downregulated genes in HF-SCs in 2-week vs 3-week *Piezo1* iKO samples. **F**, Expression levels of cell cycle-related genes, including *Cenpe*, *Cenpf*, *Mki67* and *Top2a*, in the HFSC cluster in control and *Piezo1* iKO samples. **G**, Control, 2-week and 3-week *Piezo1* iKO HFSC cluster show similar cell cycle scores.

Figure S9

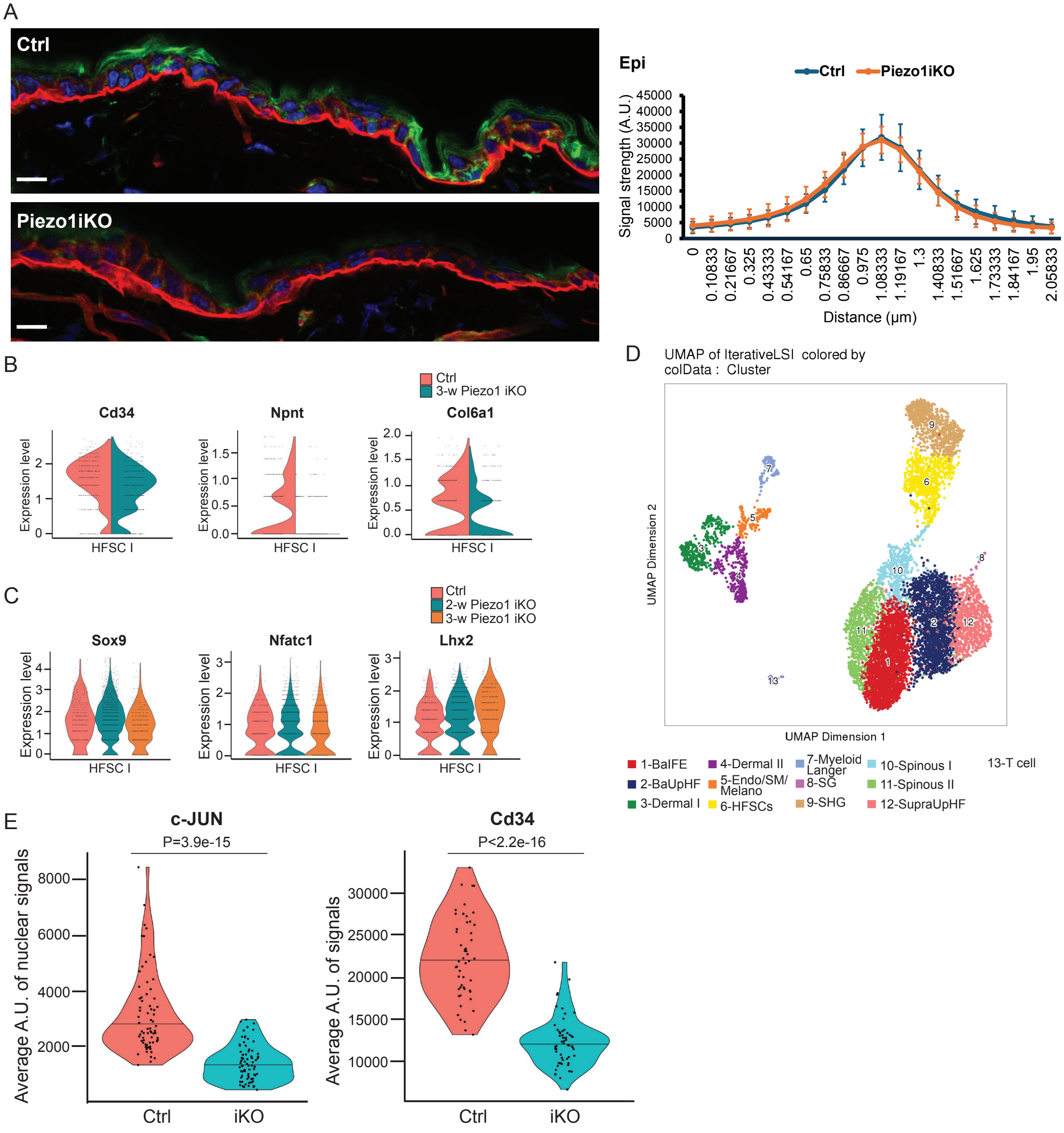
A
Ctrl
Piezo1iKO
Epi
Ctrl
Piezo1iKO
Signal strength (A.U.)
45000
40000
35000
30000
25000
20000
15000
10000
5000
0
0
0.10833
0.21667
0.325
0.43333
0.54167
0.65
0.75833
0.86667
0.975
1.08333
1.19167
1.3
1.40833
1.51667
1.625
1.73333
1.84167
1.95
2.05833
Distance (μm)
B
Ctrl
3-w Piezo1 iKO
Cd34
Npnt
Col6a1
Expression level
HFSC I
C
Ctrl
2-w Piezo1 iKO
3-w Piezo1 iKO
Sox9
Nfatc1
Lhx2
Expression level
HFSC I
D
UMAP of IterativeLSI colored by
colData : Cluster
UMAP Dimension 2
UMAP Dimension 1
1-BaIFE
2-BaUpHF
3-Dermal I
4-Dermal II
5-Endo/SM/ Melano
6-HFSCs
7-Myeloid Langer
8-SG
9-SHG
10-Spinous I
11-Spinous II
12-SupraUpHF
13-T cell
E
c-JUN
P=3.9e-15
Average A.U. of nuclear signals
8000
6000
4000
2000
Ctrl
iKO
Cd34
P<2.2e-16
Average A.U. of signals
30000
25000
20000
15000
10000
Ctrl
iKO

Fig. S9. PIEZO1 controls gene expression through transcription factor NFATC1

A, IF staining (left) and quantification (right) of α6-integrin shows no change in interfollicular epidermis after 3-week induction of *Piezo1* depletion. Scale bar, 10 μm. The error bars represent s.e.m. ($n = 15$ quantified lines). **B**, NFATC1 target genes, including *Cd34*, *Npnt* and *Col6a1*, are downregulated in 3-week *Piezo1* iKO HFSC cluster. **C**, mRNA levels of transcription factor genes *Sox9*, *Nfatc1* and *Lhx2* are not changed in *Piezo1* iKO HFSC cluster. **D**, UMAP plot of dorsal skin cell populations as detected by open chromatin landscape determined by scATACseq. **E,** Quantification of c-JUN and Cd34 signals in bulge HF-SCs in control and *Piezo1* iKO after 3-week induction. 70 bulge HF-SCs from 3 control HFs and 78 bulge HF-SCs from 3 *Piezo1* iKO HFs are quantified for c-JUN. 51 bulge HF-SCs from 3 control HFs and 57 bulge HF-SCs from 3 *Piezo1* iKO are quantified for Cd34. P values are calculated by Student's t-test.

Figure S10

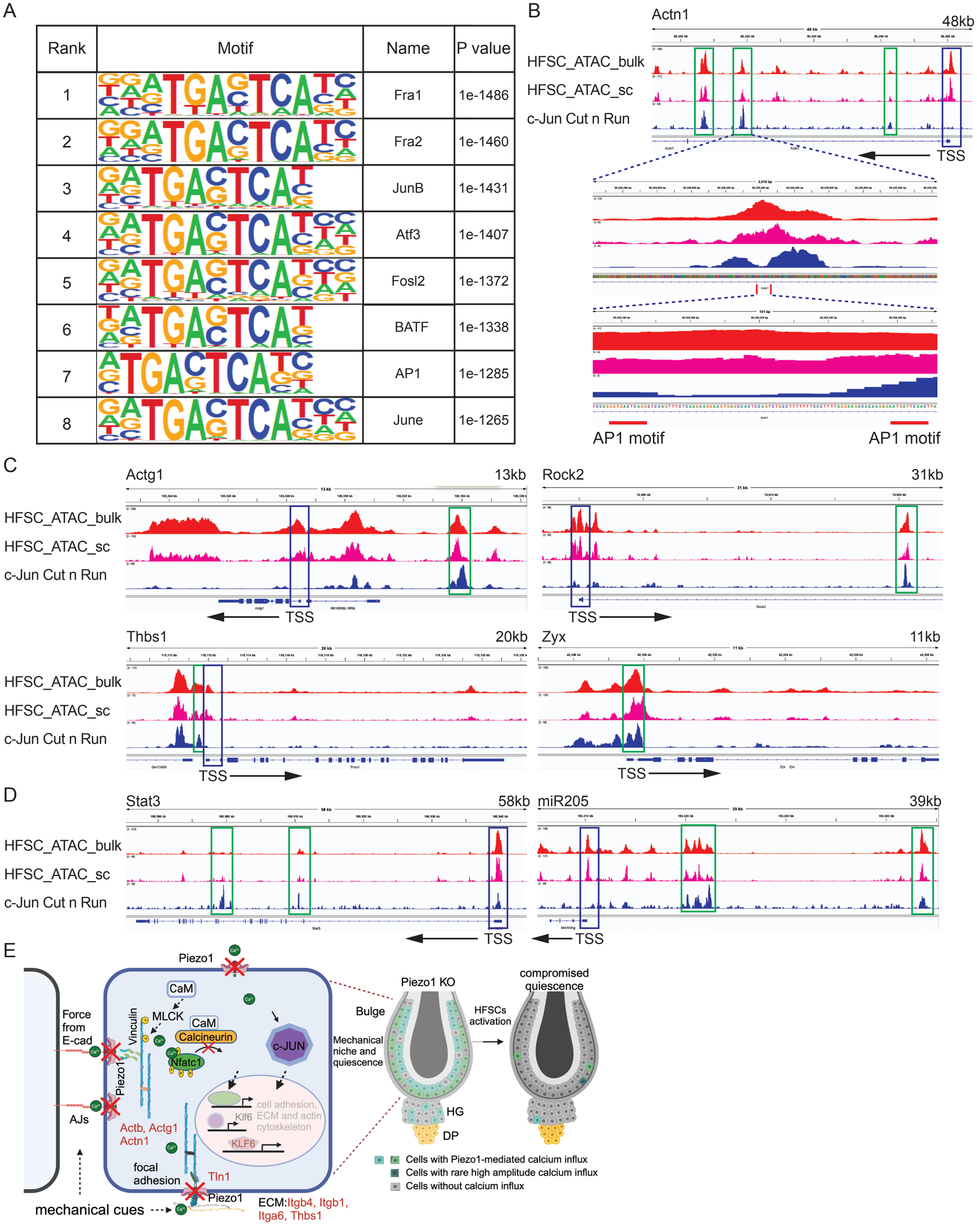

A
Rank
Motif
Name
P value
1 Fra1 1e-1486
2 Fra2 1e-1460
3 JunB 1e-1431
4 Atf3 1e-1407
5 Fosl2 1e-1372
6 BATF 1e-1338
7 AP1 1e-1285
8 June 1e-1265
B
Actn1
48kb
HFSC_ATAC_bulk
HFSC_ATAC_sc
c-Jun Cut n Run
TSS
AP1 motif
AP1 motif
C
Actg1
13kb
Rock2
31kb
HFSC_ATAC_bulk
HFSC_ATAC_sc
c-Jun Cut n Run
TSS
TSS
Thbs1
20kb
Zyx
11kb
HFSC_ATAC_bulk
HFSC_ATAC_sc
c-Jun Cut n Run
TSS
TSS
D
Stat3
58kb
miR205
39kb
HFSC_ATAC_bulk
HFSC_ATAC_sc
c-Jun Cut n Run
TSS
TSS
E
Piezo1
CaM
MLCK
CaM
Calcineurin
Nfatc1
c-JUN
Vinculin
Piezo1
Force from E-cad
AJs
Actb, Actg1
Actn1
cell adhesion, ECM and actin cytoskeleton
Klf6
KLF6
focal adhesion
Tln1
Piezo1
ECM:Itgb4, Itgb1, Itga6, Thbs1
mechanical cues
Piezo1 KO
compromised quiescence
Bulge
HFSCs activation
Mechanical niche and quiescence
HG
DP
Cells with Piezo1-mediated calcium influx
Cells with rare high amplitude calcium influx
Cells without calcium influx

Fig. S10. PIEZO1 regulates the expression of ECM, cell adhesion and actin genes through transcription factors AP1

A, AP1 motifs are the most highly enriched in c-Jun Cut & Run peaks. **B**, IGV track of bulk ATACseq, scATACseq and c-Jun Cut&Run peaks in bulge HF-SC. Note the AP1 motifs show depleted Cut & Run reads, indicating the protection of c-Jun bound region. **C-D**, Selected IGV tracks of bulk ATACseq, scATACseq and c-Jun Cut&Run peaks in bulge HF-SC for AP1 targeted genes, including actin cytoskeleton genes Actg1, Rock2, Thbs1 and Zyx (**C**) and transcription factor Stat3 and post-transcriptional regulator miR-205 (**D**). Blue box indicates the TSS, green box indicates the c-Jun binding, arrow indicates the direction of the transcription. **E,** A schematic illustration for the loss of *Piezo1* leading to compromised quiescence.

Other Supplementary Materials for this manuscript include the following:

Data S1 to S6

Movies S1 to S10

Data S1: Commonly downregulated genes shared in 2- and 3-week Piezo1 KO HF-SCs

Data S2: Commonly upregulated genes shared in 2- and 3-week Piezo1 KO HF-SCs

Data S3: Downregulated genes in Piezo1 KO HF-SCs compared with downregulated genes in Nfatc1 KO HF-SCs

Data S4: Downregulated genes detected in 3-week Piezo1 KO HF-SCs

Data S5: Downregulated genes detected in 2-week Piezo1 KO HF-SCs

Data S6: Predicted AP1 targets in commonly downregulated genes shared in 2- and 3-week Piezo1 KO HF-SCs

Movie S1: Representative calcium spikes in interfollicular epidermis. Time lapse intervals are 93 seconds for a total of 20 minutes and 21 seconds. Individual cells with calcium spike are marked by white-colored arrowheads. Coordinated calcium spikes in multiple cells are marked by purple-colored arrowheads. Hair follicle regions are outlined by red-colored circles.

Movie S2: Representative calcium spikes in telogen hair follicle. Time lapse intervals are 2 minutes for 90 minutes.

Movie S3: Representative calcium spikes in early anagen hair follicle. Time lapse intervals are 2 minutes for 90 minutes.

Movie S4: Representative calcium spikes in control hair follicles before and after Yoda1 treatment. The same hair follicle is recorded for a total of three imaging sessions. Time lapse intervals are 2 minutes for 30 minutes for each imaging session.

Movie S5: Representative calcium spikes in Piezo1 KO hair follicles before and after Yoda1 treatment. The same hair follicle is recorded for a total of two imaging sessions. Time lapse intervals are 2 minutes for 30 minutes for each imaging session.

Movie S6: Unattached microbead does not trigger calcium influx. When an unattached microbead is dislodged from the cell surface, no calcium flicker is detected. Time lapse intervals are 3 seconds.

Movie S7: Mechanical pulling of an attached microbead from the cell surface triggers a weak and localized calcium flicker. Red circle indicates the area of the microbead, yellow arrowhead indicates the calcium flicker. The same sequence is played twice. Time intervals are 3 seconds.

Movie S8: PIEZO1 inhibitor abolishes the mechanical pulling induced calcium flicker. Incubation of GsMTx4, a PIEZO1 inhibitor, blocks calcium flicker induced by the mechanical pulling. Red circle indicates the area of the microbead, yellow arrowhead indicates the lack of calcium flicker. Time lapse intervals are 3 seconds.

Movie S9: Membrane scratching triggers strong calcium spikes that are spreading to neighboring cells. Red polygon indicates the scratched cell, and yellow polygons indicate neighboring cells, which are not scratched but triggered to show strong calcium spikes within a few seconds. Note the strong nuclear calcium signals. Time lapse intervals are 3 seconds.

Movie S10: Membrane scratching induced calcium spikes are insensitive to PIEZO1 inhibitor. In the presence of GsMTx4, a PIEZO1 inhibitor, the scratching induced calcium spikes are unaffected. Time lapse intervals are 3 seconds.
